# The End of the Beginning of Mechanical Stereochemistry

**DOI:** 10.1021/acs.accounts.4c00195

**Published:** 2024-06-03

**Authors:** Stephen M. Goldup

**Affiliations:** School of Chemistry, University of Birmingham, Birmingham B15 2TT, U.K.

## Abstract

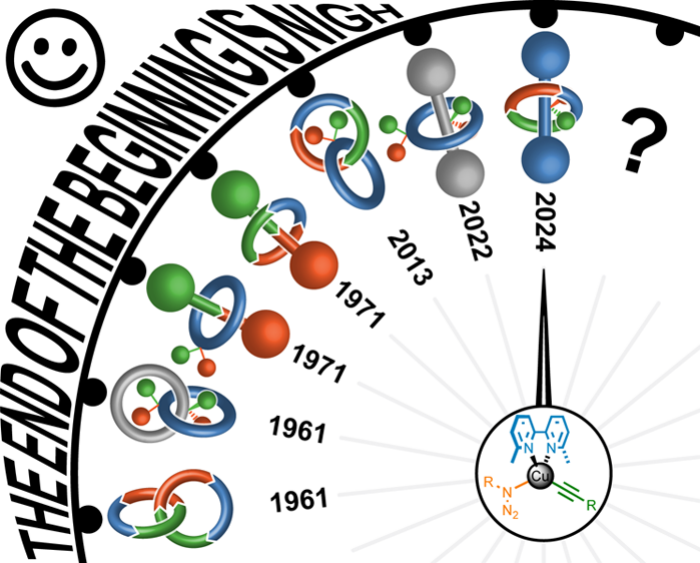

Stereochemistry has played a key role in the development of synthetic chemistry for the simple reason that the function and properties of most molecules, from medicine to materials science, depend on their shape and thus the stereoisomer used. However, despite the potential for rotaxanes and catenanes to display unusual forms of stereochemistry being identified as early as 1961, this aspect of the mechanical bond remained underexplored and underexploited; until 2014 it was only possible to access chiral rotaxanes and catenanes whose stereoisomerism is solely attributable to the mechanical bond using chiral stationary phase high performance liquid chromatography, which limited their production on scale and thus inhibited the investigation of their properties and applications. Furthermore, the stereogenic units of such molecules and analogues were often poorly described, which made it hard to fully articulate both what had been achieved in the field and what problems were left to solve. Relatively recently, methods to access rotaxanes and catenanes that display mechanical stereochemistry selectively have been developed, making these intriguing structures available for study in a range of prototypical applications including catalysis, sensing, and as chiral luminophores.

In this Account, we briefly discuss the history of mechanical stereochemistry, beginning in 1961 when the potential for mechanical stereoisomerism was first identified, before defining how mechanical stereochemistry arises from a structural point of view. Building on this, using simple stereochemical arguments, we confirm that the complete set of unique stereogenic units of two-component rotaxanes and catenanes have finally been identified and categorized unambiguously, with the last being identified only in 2024. After pausing to discuss some of the stereochemical curiosities that arise when molecules contain both covalent and mechanical stereogenic units, and the potential for stereoisomerism to arise due to co-conformational movement, we use our stereochemical framework to summarize our efforts to develop conceptually general approaches to [2]catenanes and [2]rotaxanes containing all of the possible mechanical stereogenic units. In particular, we highlight how the nature of a mechanical stereogenic unit affects the available strategies for their stereoselective synthesis. We finish by highlighting recent prototypical chemical applications of interlocked molecules that rely on their mechanical stereochemistry, before discussing future directions and challenges.

Taken together, we propose that the transition of such molecules from being hard to make and poorly described, to being available in high stereopurity using clearly articulated methodological and stereochemical concepts suggests that the field is finally maturing. Thus, we are now coming to the end of the beginning of mechanical stereochemistry. The stage is now set for such molecules to play a functional role in a range of areas, indeed in any chemical or physical application where control over molecular shape is required.

## Key References

- de Juan, A.; Lozano, D.; Heard, A. W.; Jinks, M. A.; Suarez, J. M.; Tizzard, G. J.; Goldup, S. M. A chiral interlocking auxiliary strategy for the synthesis of mechanically planar chiral rotaxanes. Nat. Chem.2022, 14 ( (2), ), 17934845345 10.1038/s41557-021-00825-9PMC7612332.^1^ A general approach to mechanically planar chiral rotaxanes that takes advantage of high diastereoselectivity and mechanical motion, demonstrated through the synthesis of 11 highly enantioenriched targets.
- Maynard, J. R. J.; Gallagher, P.; Lozano, D.; Butler, P.; Goldup, S. M. Mechanically axially chiral catenanes and noncanonical mechanically axially chiral rotaxanes. Nat. Chem.2022, 14 ( (9), ), 103835760959 10.1038/s41557-022-00973-6PMC7613450.^2^ The first stereoselective synthesis of mechanically axially chiral catenanes, which was enabled by proper stereochemical analysis that also led to the discovery of the mechanical axial stereogenic unit of rotaxanes.
- Pairault, N.; Rizzi, F.; Lozano, D.; Jamieson, E. M. G.; Tizzard, G. J.; Goldup, S. M. A catenane that is topologically achiral despite being composed of oriented rings. Nat. Chem.2023, 15 ( (6), ), 78137169983 10.1038/s41557-023-01194-1.^3^ A clear demonstration that the mechanically planar chiral stereogenic unit of catenanes is not necessarily topological in nature.
- Heard, A. W.; Goldup, S. M. Synthesis of a Mechanically Planar Chiral Rotaxane Ligand for Enantioselective Catalysis. Chem2020, 6 ( (4), ), 99432309674 10.1016/j.chempr.2020.02.006PMC7153771.^4^ The first demonstration of a mechanically planar chiral ligand in catalysis.

## Introduction

The selective synthesis of stereoisomers is a problem that continues to engage the synthetic community, driven both by the intellectual challenge it presents and the technological importance of providing stereopure molecules for applications from medicine to materials science. However, although mechanically interlocked molecules (MIMs)^5^ have attracted significant interest as components of molecular machines,^6,7^ the stereochemical properties of the mechanical bond have received less attention. This is despite there being opportunities for stereoisomerism distinct from that of classical covalently bonded molecules. Indeed, optical and geometric isomerism can arise in MIMs even when their covalent subcomponents are stereochemically trivial because of how the underlying symmetry properties of the subcomponents interact in the geometrically restricted environment of the mechanical bond.^8^

In this Account, we provide an overview of how the study of molecules displaying mechanical stereochemistry has progressed since the first racemic syntheses of mechanically chiral molecules in the 1990s. We also discuss how our understanding of mechanical stereochemistry has evolved during these synthetic efforts. Our focus is on the stereochemistry that arises in molecules composed of two covalent subcomponents (e.g., [2]catenanes and [2]rotaxanes) that contain the minimum number of crossing points for a mechanical bond to exist. We conclude with a discussion of the next frontiers of mechanical stereochemistry, from the possible applications of these molecules to future synthetic challenges.

## Describing Mechanical Stereochemistry

### The Canonical Mechanical Stereogenic Units

**Figure 1:**
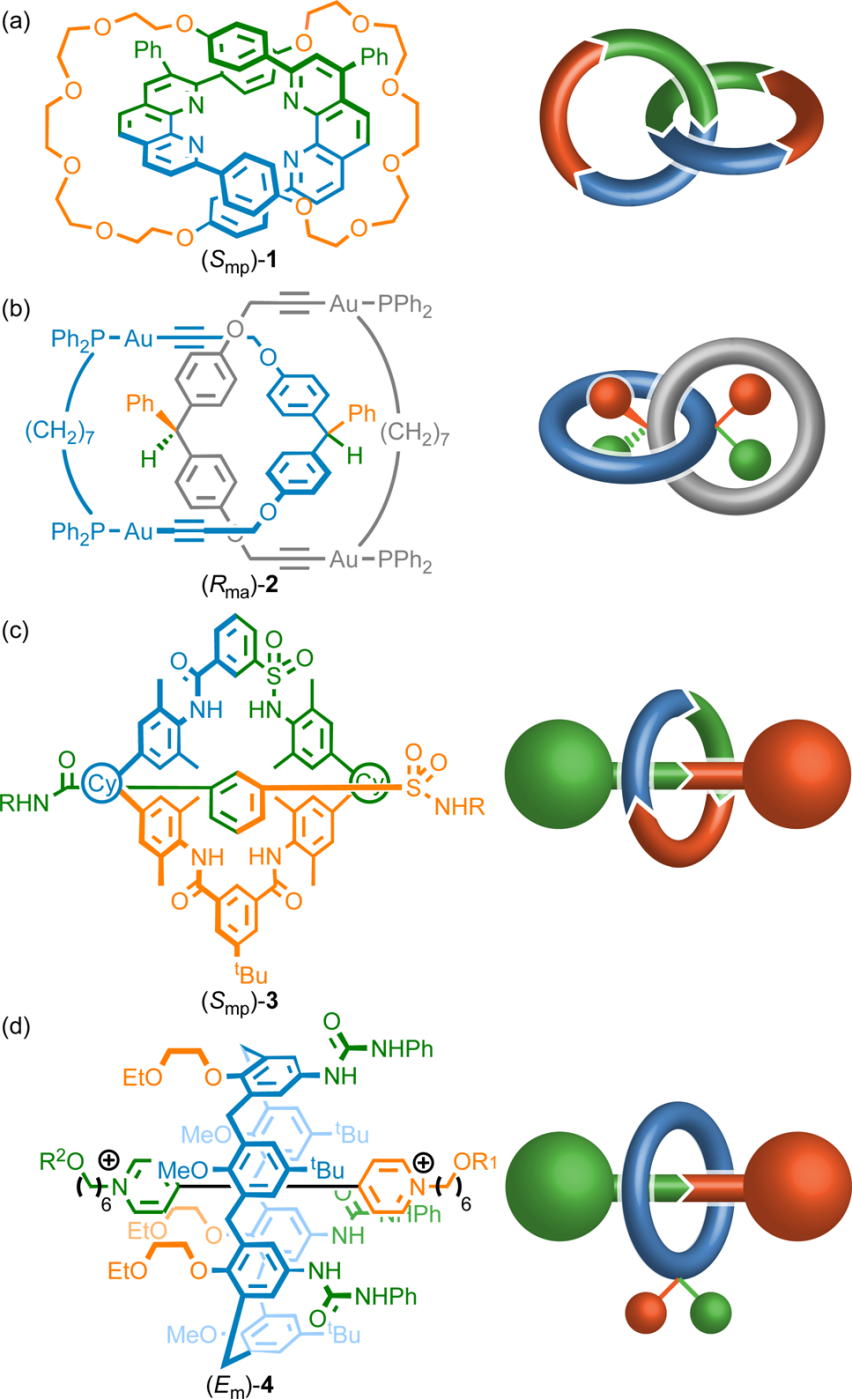
The canonical mechanical stereogenic units identified by Wassermann and Frisch^9^ (a, b) and Schill^11^ (c, d) and representative early chemical examples of such structures which were separated by PCSP-HPLC (**1**^13^ and **3**(14) [R = 4-C_6_H_4_CPh_3_; Cy = 1,1-cyclohexyl]), reported as a racemate (**2**^18^) or synthesized stereoselectively (**4**^16^ [R^1^ = C(O)CH(cyclohexyl)_2_; R^2^ = C(O)CHPh_2_]).^10^

In 1961,^9^ Wasserman and Frisch highlighted that chirality could arise in catenanes even when the component rings are achiral provided that the two rings are oriented by a sequence of atoms (Figure 1a^10^), or the two faces of the macrocycles are distinguishable (Figure 1b). Later,^11^ Schill recognized that chiral rotaxanes arise if the macrocycle is oriented and the two ends of the axle are distinguishable (Figure 1c) and also that geometric isomerism arises if the macrocycle faces and the two ends of the axle are distinguishable (Figure 1d). We later described such molecules as displaying “conditional mechanical stereochemistry” because their stereochemistry is conditional on the symmetry of the subcomponents.^8a^

Molecules whose stereochemistry relies solely on these canonical stereogenic units received varying levels of attention between their initial identification^9^ and 2014^12^ when we made our first contribution. Specifically, the enantiomers of chiral catenanes and rotaxanes (Figure 1) composed of oriented components (e.g., **1**(13) and **3**(14) respectively) had been separated using preparative chiral stationary phase HPLC (PCSP-HPLC)^15^ and several stereoselective syntheses of rotaxane geometric isomers had been reported, albeit these relied on the use of macrocycles that adopt a cone-shaped conformation, typically calixarenes (e.g., **4**(16)), rather than simple prochiral^17^ rings as envisaged by Schill.^11^ However, no report of enantioenriched catenanes composed of facially dissymmetric rings (e.g., **2**(18)) had been disclosed.

In 2011,^19^ we observed well-expressed diastereomerism in sterically crowded rotaxanes containing covalent stereochemistry. This observation prompted us to develop auxiliary methodologies for the stereoselective synthesis of mechanically stereogenic molecules. Our guiding objective was to be able to synthesize structures where the mechanical bond provides the sole source of stereoisomerism to allow the potential applications of mechanical stereochemistry to be identified unambiguously. In addition to synthetic challenges, our studies revealed problems relating to the description of mechanical stereochemistry, as highlighted by the relatively recent identification of noncanonical chiral^2^ and geometric^20,21^ stereogenic units. Thus, we have also worked to systematize the description of mechanical stereochemistry—in order to know if we have achieved our goal, we need to know how many stereogenic units there are to conquer!

### Defining the Fundamental Mechanical Stereogenic Units

**Figure 2:**
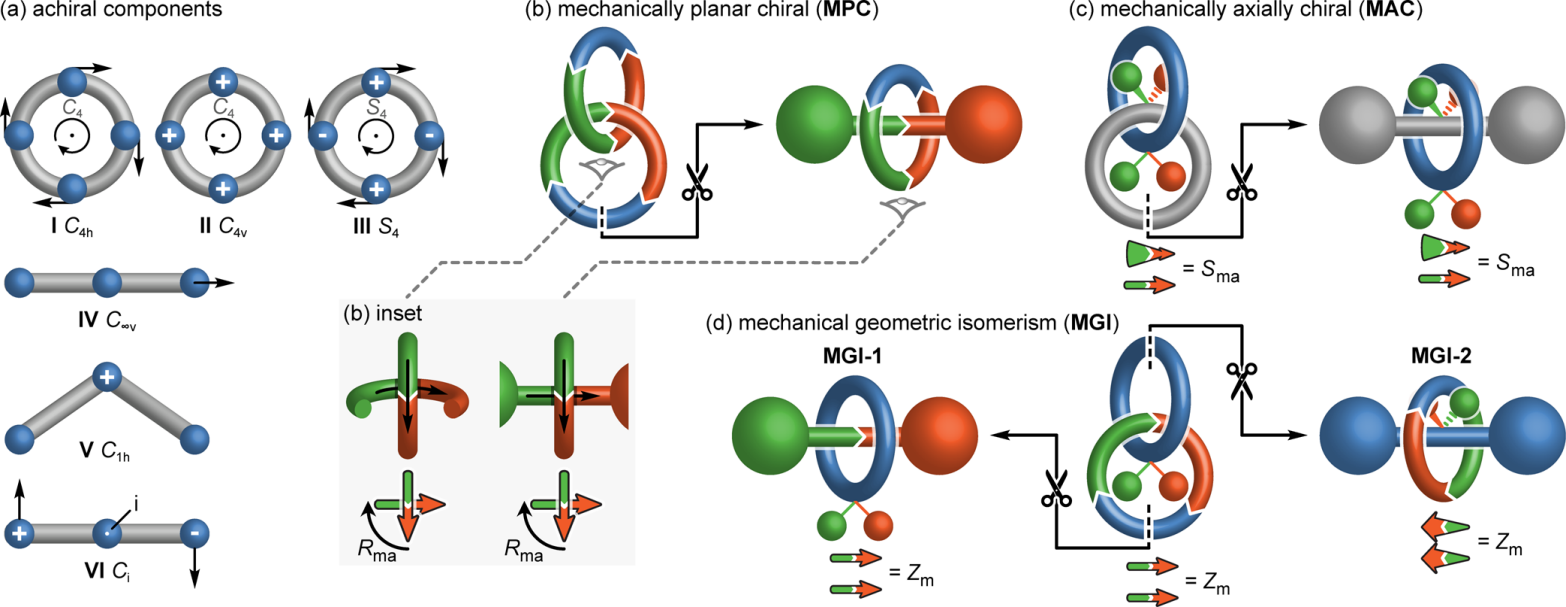
(a) Achiral ring (**I**–**III**) and axle (**IV**–**VI**) components that give rise to (b) MPC (inset shows the view used to assign the mechanical stereochemistry), (c) MAC, and (d) MGI stereochemistry.^10^

The stereoisomers of molecules displaying mechanical stereochemistry are related by inversion of the relative orientation of the two interlocked components. We recently highlighted^21^ that such isomerism can only arise when neither covalent subcomponent contains a *C*_2_ rotational axis parallel to the macrocycle plane/perpendicular to the axle long axis as this rotation corresponds to the notional process of inverting the orientation of the MIM components; if this rotation is a symmetry operation of the separated component it precludes mechanical steroisomerism. In hindsight this requirement should have been obvious but, to our knowledge, it had not been stated previously. The only achiral macrocycle point group symmetries that meet this requirement are *C*_*n*__h_, *C*_*n*__v_, *S*_2*n*_, which allowed us to confirm that the complete set of mechanical stereogenic units in catenanes had already been identified; *C*_*n*h_ symmetric macrocycles (**I**, Figure 2a) are by definition oriented and *C*_*n*__v_ symmetric rings (**II**) are facially dissymmetric and so catenanes composed of pairs of rings of these symmetries correspond to the chiral catenanes identified by Wasserman and Frisch,^9^ whereas interlocking one *C*_*n*__h_ symmetric ring with one *C*_*n*__v_ ring produces the geometric stereogenic unit identified more recently by Gaeta and Neri.^20^ The only surprise arising from this analysis is that *S*_2*n*_ rings also give rise to mechanical stereochemistry. However, inspecting a simple representation of an *S*_4_ symmetric structure (**III**) reveals that these rings are also oriented; *S*_2*n*_ symmetry arises when the ring is oriented, but the horizontal mirror symmetry is lifted.

Thus, we define the three conditional mechanical stereogenic units of catenanes as arising when two oriented (*C*_*n*__h_ or *S*_2*n*_, Figure 2b) or facially dissymmetric (*C*_*n*v_, Figure 2c) rings, or one oriented and one facially dissymmetric ring (Figure 2d) are combined. In the latter case, the structure is achiral as the stereochemistry is characterized by oriented lines (vectors) that can be arranged coplanar in a *syn* or *anti* arrangement. We describe such catenanes as displaying mechanical geometric isomerism (MGI). The stereochemistry arising when two facially dissymmetric rings are combined is characterized by vectors perpendicular to each ring that cannot be made coplanar, whereas the equivalent vectors arising from interlocked oriented rings are parallel to the macrocycle plane. The former are usually termed “mechanically axially” chiral (MAC) catenanes^5^ whereas the latter have historically been simply referred to as “topologically chiral” catenanes because their stereochemistry is a topological property^22^ of the structure when the orientation arises from a sequence of atoms within the macrocycle. However, we have suggested that “mechanically planar” chiral (MPC) is more appropriate because this stereochemistry can also be Euclidean^3^ and the catenane and rotaxane stereogenic units, to which this label was originally applied,^15,12^ are related by a notional ring opening process (Figure 2b).

It is relatively trivial to perform the same first-principals stereochemical analysis for rotaxanes, with the result that mechanical stereochemistry arises in rotaxanes if the axle is oriented (*C*_*n*__v_ symmetry, e.g., **IV**) or facially dissymmetric (*C*_*n*__h_ or *C*_i_ symmetry, e.g., **V** or **VI** respectively).^21^ However, it is more intuitive to recognize that rotaxanes and catenanes are related by a notional ring-opening-and-stoppering process. Applying this approach, MPC (Figure 2b) and MAC (Figure 2b) catenanes are each related to a chiral rotaxane mechanical stereogenic unit and thus we have proposed that the same nomenclature be used to rotaxane and catenane stereochemistry. The MPC rotaxane stereogenic unit corresponds to that originally proposed by Schill,^11^ whereas MAC rotaxanes were overlooked until 2022.^2^

Finally, if the oriented ring of an MGI catenane is chosen for the ring opening process (Figure 2d), the rotaxane product displays geometric isomerism, which corresponds to that first identified by Schill.^11^ However, if the facially dissymmetric ring is opened, the corresponding rotaxane displays a form of geometric isomerism that we have only recently highlighted.^21^ We proposed that the labels “type 1 MGI” (MGI-1) and “type 2 MGI” (MGI-2) be applied to disambiguate these forms or rotaxane stereoisomerism, where the numeral simply refers to the order of their identification.

### Assigning Mechanical Stereochemistry

Given that the stereoisomers of rotaxanes and catenanes are clearly related by a notional ring opening process, it seems sensible that (i) the method used to assign their absolute stereochemistry should avoid automatic inversion of the stereolabel when this process is considered and (ii) the methods used should require the minimum number of arbitrary rules. We recently fell afoul of these rules; our proposed method for assigning the stereochemistry of axially chiral rotaxanes could not be extended to type 2 rotaxane geometric isomers without causing an inversion of stereolabel compared with the corresponding catenane.^23^ To overcome this problem, we have revised our methods for assigning the axial stereogenic unit of rotaxanes and catenanes.

Pleasingly, this change resulted in a fully self-consistent approach to assigning the fundamental mechanical stereogenic units. In broad terms (see ESI for full details) one: (i) defines the vector associated with each component using simple rules based on the Cahn-Ingold-Prelog^24^ priorities of atoms; (ii) views these vectors at the crossing point between the two components (Figure 2b inset); (iii) if the vectors cannot be made coplanar, identifies if the direction of rotation from the head of the front vector to the tail of the rear vector is clockwise (*R*) or anticlockwise (*S*); (iv) if the vectors can be made coplanar, identifies if they are parallel (*Z*) or antiparallel (*E*). We propose that the suffixes “mp”, “ma” and “m” be applied to the stereolabels for MPC, MAC and MGI structures respectively (e.g., *R*_mp_, *R*_ma_ and *Z*_m_) to indicate the mechanical origin of the stereoisomerism.^25^

### Molecules Containing Covalent and Mechanical Stereogenic Units

**Scheme 1:**
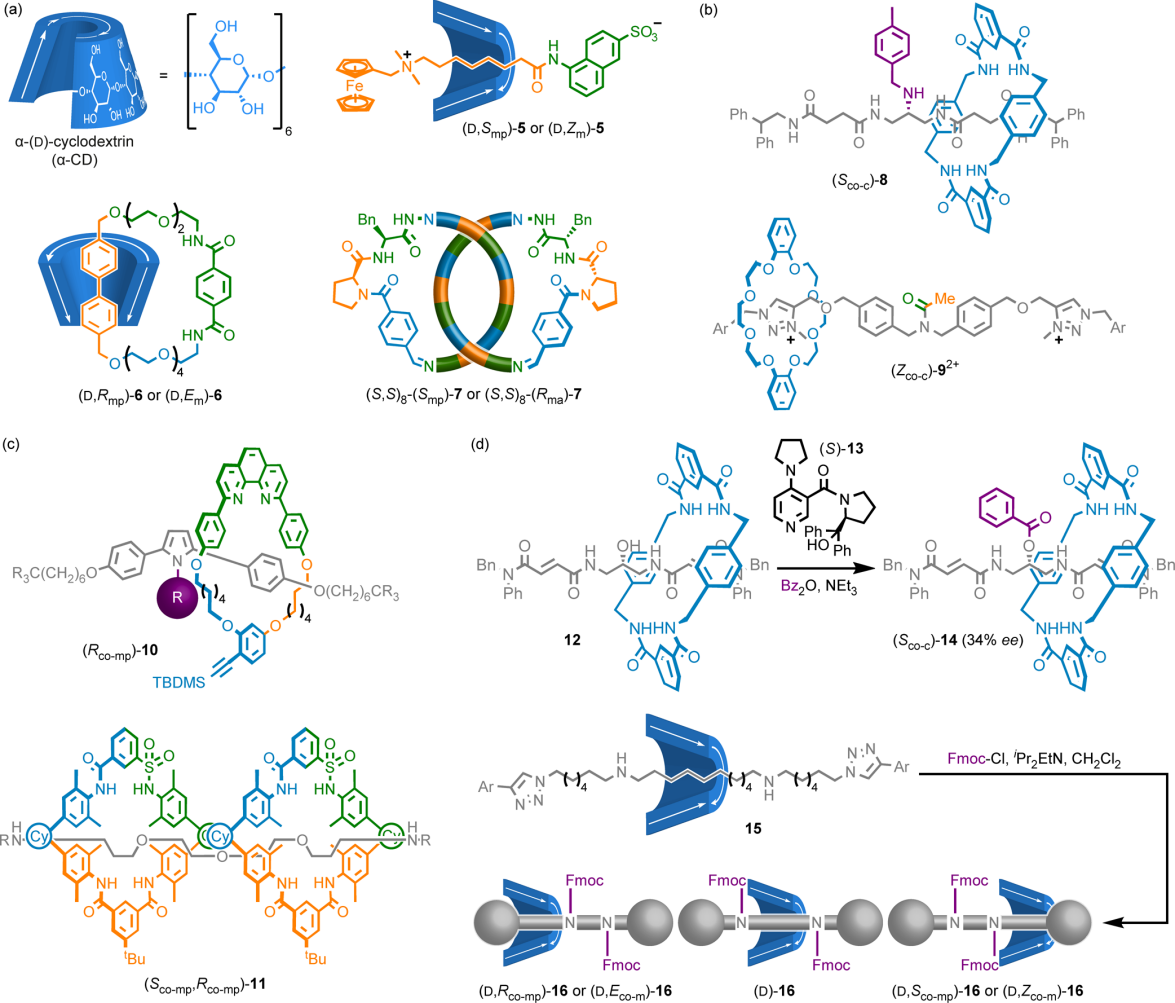
(a) MIMs Containing Covalent Stereocenters Whose Stereochemistry can be Described Using One of Two Mechanical Stereodescriptors. (b) MIMs that Display Co-conformational Covalent Stereochemistry (Ar = 3,4-di-^*t*^Bu-C_6_H_3_). (c) MIMs that Display Co-conformational MPC Stereochemistry (R^1^ = 4′-cyclohexyl-1,1′-biphenyl, R^2^ = C(O)-(4-C_6_H_4_)–CH_2_O-(4-C_6_H_4_)-CPh_3_). (d) Operation of Information Ratchets **12** and **16** (Ar = 3,4-di-^*t*^Bu-C_6_H_3_)^10^

Many mixed covalent/mechanical diastereomers have been reported.^8a^ However, due to difficulties in clearly describing their stereochemistry, the importance of these early results was often obscured. Most commonly, these molecules contain cyclodextrin (CD) rings (Scheme 1a), which are both oriented and facially dissymmetric, as well as containing covalent stereogenic units and so rotaxane **5**(26) and catenane **6**(27) exist as mechanical diastereomers. Such molecules were often described as “orientational” isomers. However, all mechanical stereochemistry depends on the relative orientation of the subcomponents.

MIMs containing mechanical and covalent stereochemistry can typically be fully specified using one of two possible mechanical stereodescriptors combined with the relevant covalent stereolabels. For example, the mechanical stereochemistry of **5** and **6** can be specified as MGI or MPC but using both labels is redundant because one automatically specifies the other. We hesitate to arbitrarily privilege one description over the other. However, in the case of catenanes **6** the MPC label seems more appropriate as this captures a key feature of the stereochemistry; although MGI stereochemistry is not a topological property of the structure, the MPC stereochemistry is. For this reason, we prefer the covalent-MPC description of **6** and, for consistency, apply the same approach to rotaxanes **5**. Similarly, the configuration of peptidic catenane **7**(28) can be fully specified using a MAC or MPC stereolabel alongside the covalent configuration, of which we prefer the MPC description for the same reason as above.

### Co-conformational Stereochemistry

We have so far focused on conditional mechanical stereochemistry that is invariant with mechanical motion. However, co-conformational movement can result in new stereogenic units. In the simplest case, the position of one subcomponent desymmetrizes the other such that covalent co-conformational stereochemistry arises (Scheme 1b), as in enantioselective catalyst **8**,^29^ which is chiral because the ring desymmetrizes an axle covalent prochiral center that is bulky enough to prevent racemization (*c.f.*, atropisomerism). Similarly, rotaxane **9** displays dynamic co-conformational covalent geometric isomerism as the amide geometric isomers can exchange by shuttling of the macrocycle either side of the amide bond as well as by single bond rotation.^30^

Co-conformational mechanical stereochemistry arises when one component desymmetrizes the other (Scheme 1c). Rotaxane **10** displays co-conformational MPC stereochemistry as the position of the oriented ring desymmetrizes the axle component.^31^ Similarly, rotaxane **11** exists as two non-interchanging^32^ co-conformational diastereomers, one of which is chiral and the other is *meso* (shown) because the oriented rings desymmetrize the bilaterally symmetrical axle.^33^

Co-conformational stereochemistry has been harnessed to generate directed mechanical motion (Scheme 1d). Rotaxane **12** behaves as a molecular information ratchet under enantioselective acylation of the OH unit mediated by (*S*)-**13**.^34^ Similarly, information ratchet **15** relies on the fact that shuttling of the ring results in mixed covalent/co-conformational epimeric structures that react at different rates with FmoCl,^35^ resulting in kinetic asymmetry^36^ and thus a mixture of rotaxanes **16** in ratios that do not accord with their relative stability.

## Auxiliary Syntheses of MPC Structures

**Scheme 2:**
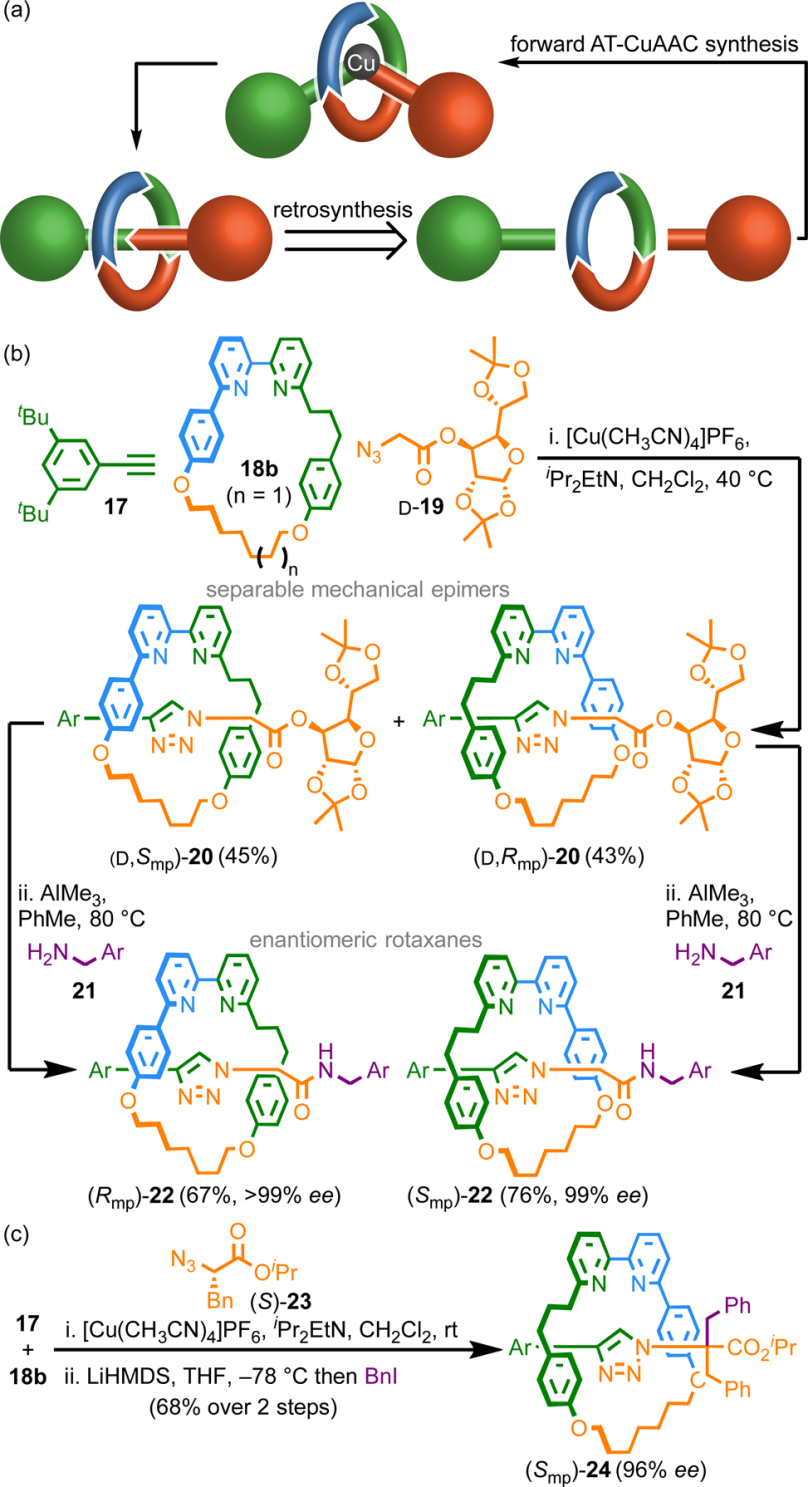
(a) Retrosynthesis Demonstrating that Dividing the Axle or Macrocycle Component of an MPC Rotaxane Results in Achiral Components. (b) A Chiral Derivatization Approach to MPC Rotaxanes **22**. (c) A Stereoselective Chiral Auxiliary Approach to Rotaxanes **24**. Ar = 3,4-di-^*t*^Bu-C_6_H_3_^10^

The properties of the underlying stereogenic units in rotaxanes and catenanes feeds directly into methods for their synthesis.^37^ For example, if the axle or macrocycle components of an MPC rotaxane are disconnected the resulting components are always achiral (Scheme 2a). Thus, additional chiral information needs to be included in the forward synthesis if we are to avoid producing a racemic mixture of enantiomeric products.

In 2014,^12^ we demonstrated one solution to this problem through an active template^38^ Cu-mediated alkyne–azide cycloaddition^39^ (AT-CuAAC^40^) coupling between alkyne **17** and glucose-derived azide (d)-**19** mediated by readily available^41^ oriented macrocycle **18b** to produce rotaxanes **20** (Scheme 2b). Diastereomeric rotaxanes **20** were separated by flash chromatography and the covalent stereochemistry removed by aminolysis to give rotaxanes **22** in high stereopurity. Luckily, both (d-*R*_mp_)-**20** and (d-*S*_mp_)-**20** could be analyzed by single-crystal X-ray diffraction (SCXRD), which allowed their absolute stereochemistry to be assigned unambiguously, the first time this had been achieved for an MPC rotaxane. Indeed, throughout the discussions below, the absolute configuration of the mechanical stereogenic unit, where provided, was assigned using SCXRD. However, we have shown that it is possible to computationally model, and thus predict, the vibrational circular dichroism spectra of MPC rotaxanes.^62^ Although computationally expensive at present, in future, this may provide a method to assign the mechanical configuration of molecules without the need to generate crystals suitable for SCXRD.

The AT-CuAAC reaction is particularly beneficial for the synthesis of separable mechanical epimers as it is efficient even when forming highly sterically crowded products.^19^ This crowding ensures that the covalent and mechanical stereogenic units interact strongly, leading to well-expressed diastereoisomerism. The same steric hindrance is also potentially beneficial when considering stereoselective mechanical bond formation. Indeed, in 2018 we extended our approach to the first true chiral auxiliary synthesis of an MPC rotaxane by using α-chiral azide (*S*)-**23** as the auxiliary (Scheme 2c).^42^ High stereoselectivity (96% *de*) was observed when aryl acetylene **17** was the coupling partner, although selectivities fell when less hindered alkynes were used. Subsequent alkylation of the stereocenter removed the covalent stereochemistry, giving rotaxane (*S*_mp_)-**24** in 96% *ee* without separation of the intermediate diastereomers.

**Scheme 3:**
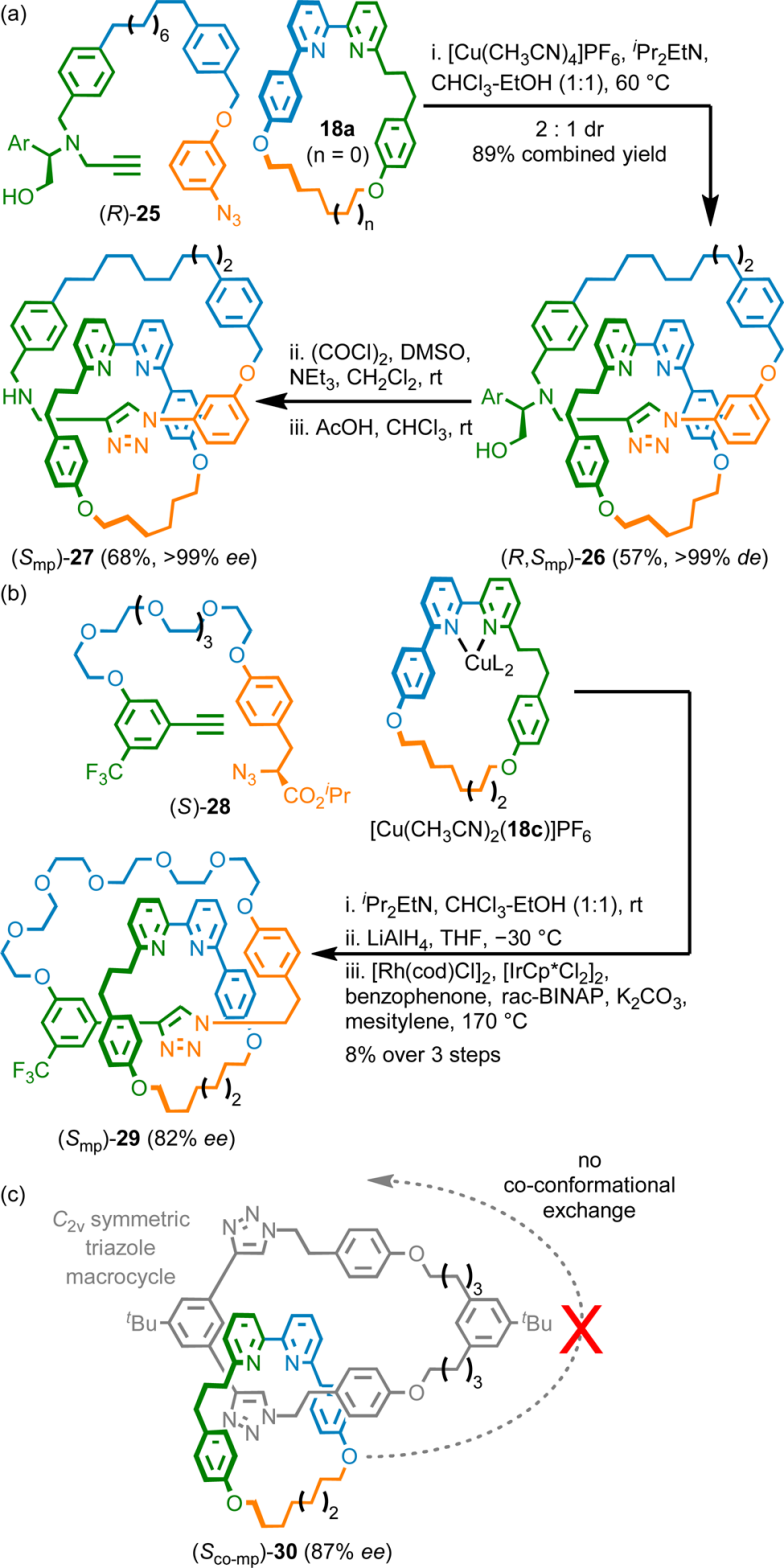
(a) Synthesis of MPC Catenane **27** (Ar = 4-OMe-C_6_H_4_). (b) Synthesis of MPC Catenane **29**. (c) Co-conformationally MPC Catenane **30**(10)

The same stereochemical considerations apply to MPC catenanes; disconnection of either ring gives rise to achiral starting materials, requiring the inclusion of a temporary source of stereochemical information in the forward synthesis. In our first report, an exocyclic pendant auxiliary was used to direct the formation of the mechanical bond in low (50% *de*) stereoselectivity (Scheme 3a).^43^ After separation of diastereomers **26**, the pendant auxiliary could be removed by oxidation-tautomerization-hydrolysis sequence to give enantiopure catenane **27**. More recently, we extended our α-chiral azide-type auxiliary to the synthesis of MPC catenane **29** in up to 82% *ee* (Scheme 2b).^44^ In this case we removed the covalent stereocenter by Rh-mediated decarbonylation. Although this sequence proceeded in low isolated yield, in part due to several challenging purifications, we were able to use the same method to synthesize co-conformationally mechanically chiral catenane (*S*_co-mp_)-**30** in high stereopurity by installing the mechanical bond selectively over one side of a C_2v_ ring (Scheme 2c).

**Scheme 4:**
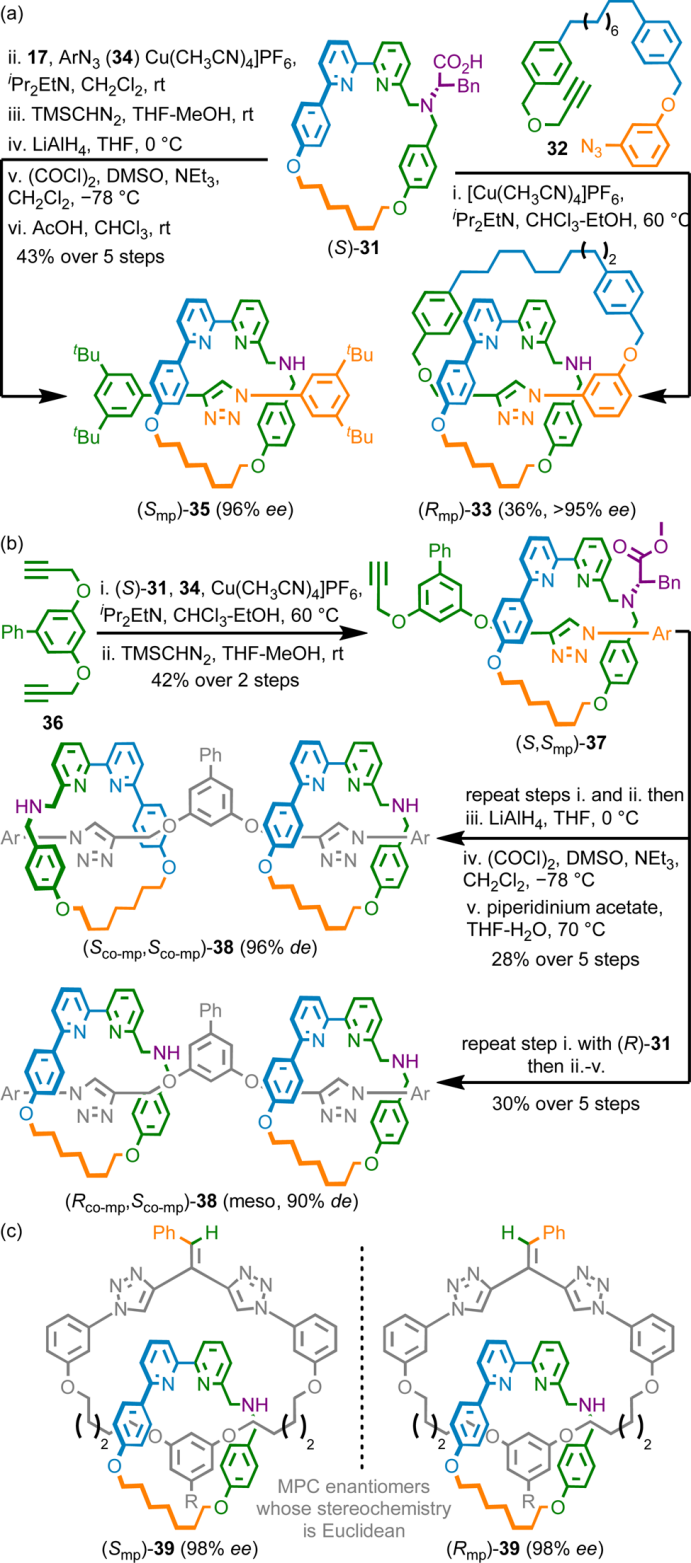
(a) MPC Catenane **33** and Rotaxane **35** Synthesized from Macrocycle (*S*)-**31** (Ar = 3,4-di-^*t*^Bu-C_6_H_3_). (b) Synthesis of Co-conformational MPC [3]Rotaxanes **38** (Ar = 3,4-di-^*t*^Bu-C_6_H_3_). (c) MPC Catenane **39** Whose Stereochemistry is Euclidean (R = CH_2_OH)^10^

Our AT-CuAAC approach to MPC rotaxanes and catenanes all make use of a bipyridine macrocycle motif, which led us to investigate whether it was possible to direct stereoselective formation of MPC rotaxanes and catenanes using a single chiral macrocycle.^45^ Pleasingly, macrocycle (*S*)-**31** was found to produce both catenanes (e.g., **33**) and rotaxanes (e.g., **35**) in very high stereoselectivity (Scheme 4a). The auxiliary moiety was removed by reduction of the carboxylic acid followed by an oxidation-tautomerization-hydrolysis sequence (*c.f*. **29**) in the case of the reported rotaxanes whereas the auxiliary cleaved spontaneously during the mechanical bond forming step in the synthesis of **33**. The origin of the latter serendipitous reactivity is as yet unknown. This flexible methodology also allowed the synthesis of all three diastereomers (*meso* and both enantiomers) of co-conformationally MPC [3]rotaxanes **38** (Scheme 4b) in which a centrosymmetric axle is encircled by two identical oriented macrocycles, simply by varying the enantiomer of macrocycle **31** used during an iterative^46^ AT-CuAAC strategy.^45^

We used the same methodology to synthesize MPC catenane **39** whose stereochemistry is Euclidean in nature, the first example of such a structure, in high stereopurity (Scheme 4c).^3^ The triazole-containing ring of **39** is oriented by the exocyclic double bond and so catenane **39** contains an MPC stereogenic unit. However, the stereochemistry of **39** is not a topological property of the structure because double bond geometry is not defined in the corresponding molecular graph.^22^

**Scheme 5:**
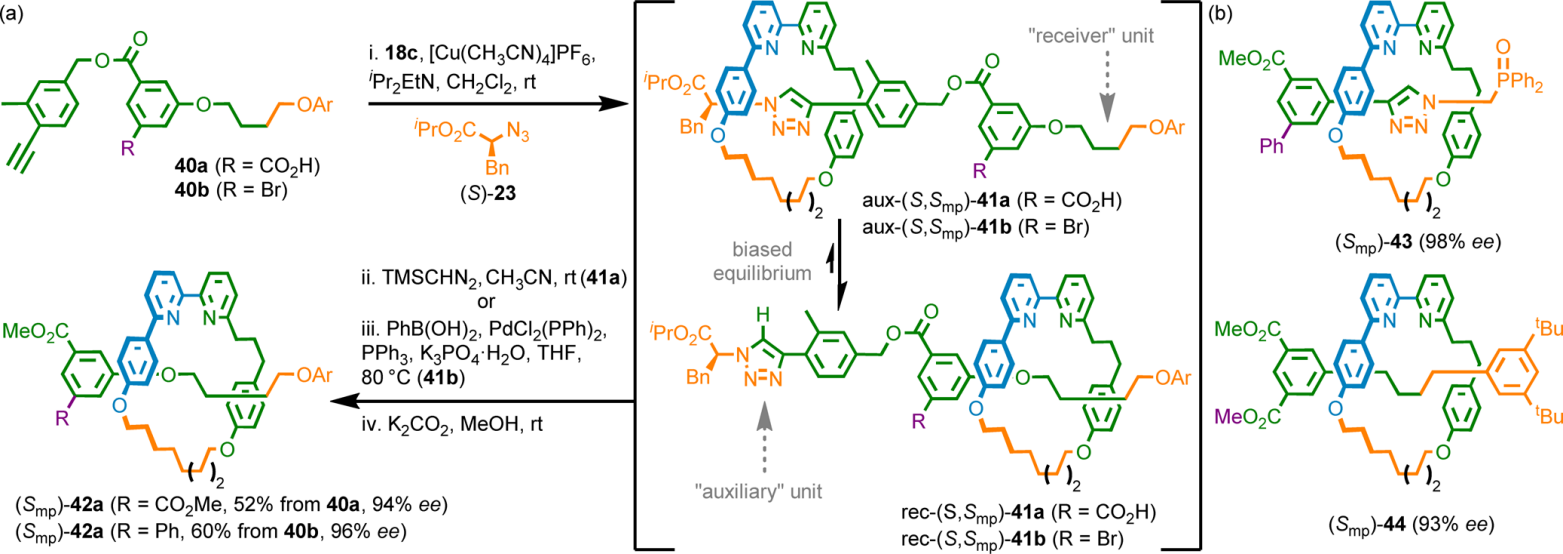
(a) Interlocking Chiral Auxiliary Synthesis of MPC Rotaxanes **42** (Ar = 3,4-di-^*t*^Bu-C_6_H_3_). (b) Rotaxanes **43** and **44** Made Using the Chiral Interlocking Auxiliary Approach^10^

Finally, as commented above, the selectivity with α-chiral azide-based auxiliaries (e.g., **23**) depends strongly on the steric demand of the alkyne coupling partner.^42^ During investigations of this effect, we serendipitously identified that an *o*-Me aryl acetylene motif delivered both high diastereoselectivity in combination with azide **23**, and resulted in products where the bipyridine macrocycle is displaced from the triazole formed, presumably due to the same steric hindrance that ensures high *de* in the mechanical bond formation.^1^ We took advantage of this observation to develop a chiral interlocking auxiliary strategy, as exemplified in the synthesis of rotaxanes **42** (Scheme 5a). Coupling of (*S*)-**23**, **18c** and alkynes **40** gave rotaxanes **41** that exist as a mixture of co-conformations in which the macrocycle preferentially encircles the alkyl ether receiver unit where it presumably engages in CH···N H-bonds with the polarized axle ether protons. Subsequent esterification (rotaxane **41a**) or cross coupling (rotaxane **41b**) traps the macrocycle over the ether receiver unit after which transesterification with basic MeOH removes the auxiliary unit to give rotaxanes **42** in excellent stereopurity (94% and 98% *ee* respectively). To demonstrate the power of this approach we synthesized 11 highly enantioenriched (93–98% *ee*) MPC rotaxanes, including (Scheme 5b) functionalized (e.g., **43**) and extremely challenging examples where the receiver unit presents almost no attractive interactions for the macrocycle (e.g., **44**).

## Syntheses of MAC Rotaxanes and Catenanes

**Scheme 6:**
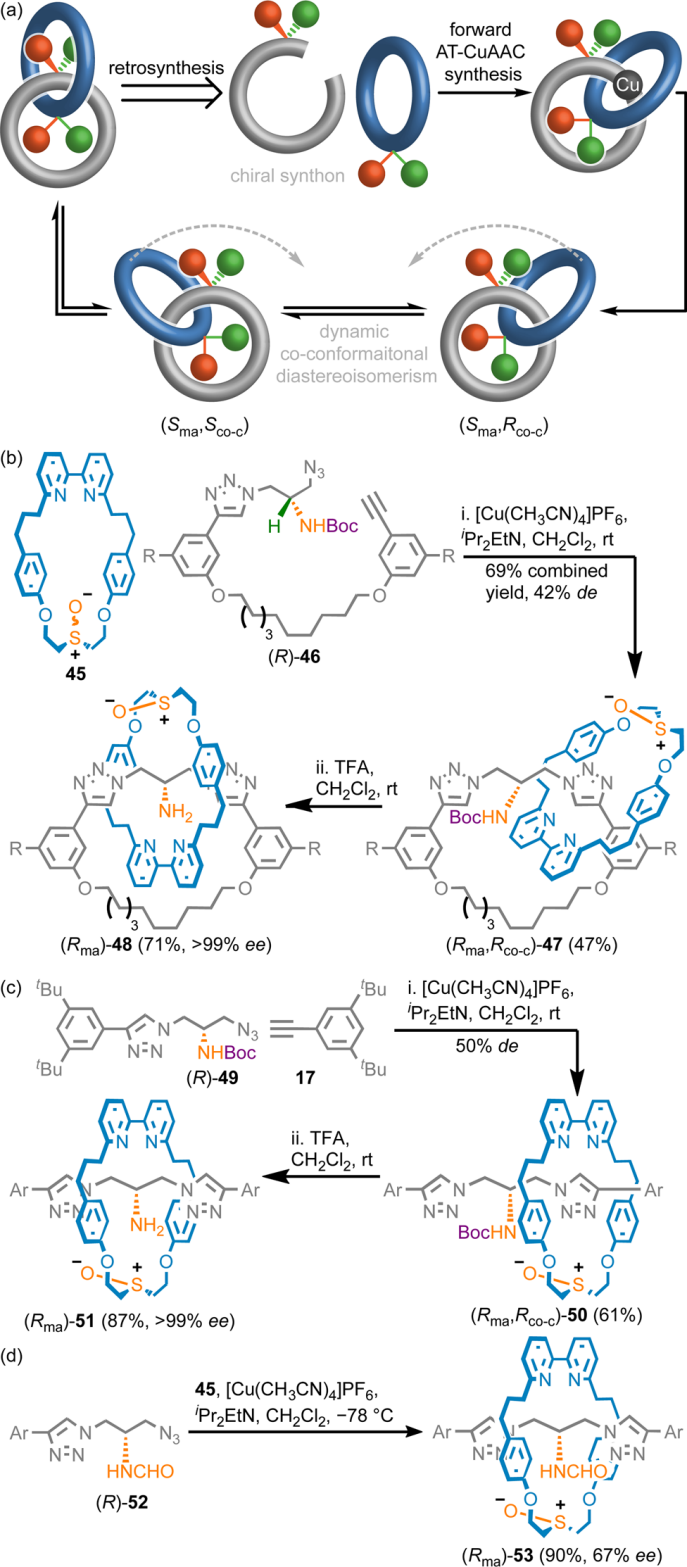
(a) Schematic Retrosynthesis of a MAC Catenane via a Chiral Synthon that is Symmetrized in the Forward Reaction Highlighting the Potential for Co-conformational Diastereoisomerism. (b) Synthesis of MAC Catenane **48** (R = CO_2_Me). (c) Synthesis of MAC Rotaxane **51** (Ar = 3,4-di-^*t*^Bu-C_6_H_3_). (d) Direct Enantioselective Synthesis of MAC Rotaxane **53** (Ar = 3,4-di-^*t*^Bu-C_6_H_3_)^10^

A key difference between MPC and MAC stereochemistry is that in the latter case, disconnecting one of the prochiral subunits results in a chiral fragment that, in the forward reaction, is symmetrized (Scheme 6a).^47^ We combined this observation with the recognition that MAC molecules display co-conformational covalent stereochemistry. If the relative movement of the rings is sterically hindered this results in separable diastereomers. The challenge then becomes controlling which face of the incoming prochiral macrocycle interacts with which component of the half-axle/pre-macrocycle.

Coupling of prochiral macrocycle **45** with chiral pre-macrocycle (*R*)-**46** gave diastereomeric catenanes **47** (42% *de*) that differ in the relative orientation of the rings–the SO unit can point toward or away from the NHBoc substituent–but whose co-conformational stereochemistry is fixed by the method of synthesis (Scheme 6b).^2^ Separation of stereoisomers **47** followed by removal of the Boc group from the major (*R*_ma_,*R*_co-c_)-**47** isomer gave MAC catenane (*R*_ma_)-**48** in excellent stereopurity (>99% *ee*). Similarly, half-axle (*R*)-**49** was coupled with macrocycle **45** to give diastereomers **50** in 50% *de* (Scheme 6c). These were separated and the Boc group removed to give MAC rotaxane (*R*_ma_)-**51** in excellent stereopurity (>99% *ee*).

Recently,^23^ we revisited the synthesis of rotaxanes **50**. The results obtained when the N protecting group and reaction conditions were varied led us to propose that the observed selectivity arises due to an NH···O interaction between the NH unit of the axle and the sulfoxide moiety of the macrocycle during the mechanical bond forming step. Under optimized conditions (CH_2_Cl_2_, −78 °C) we were able to increase the stereoselective formation (*R*_ma_)-**50** (80% *de*). This understanding allowed us to develop a direct enantioselective synthesis of MAC rotaxane **53** (Scheme 6d) in which the NCHO substituent is too small to prevent exchange of the macrocycle between prochiral compartments. Thus, the MAC stereogenic unit is the only fixed source of stereoisomerism in **53**. Coupling of formamide half-axle (*R*)-**52** with **45** and **17** at low temperature resulted in rotaxane (*R*_ma_)-**53** in up to 67% *ee*.

**Scheme 7:**
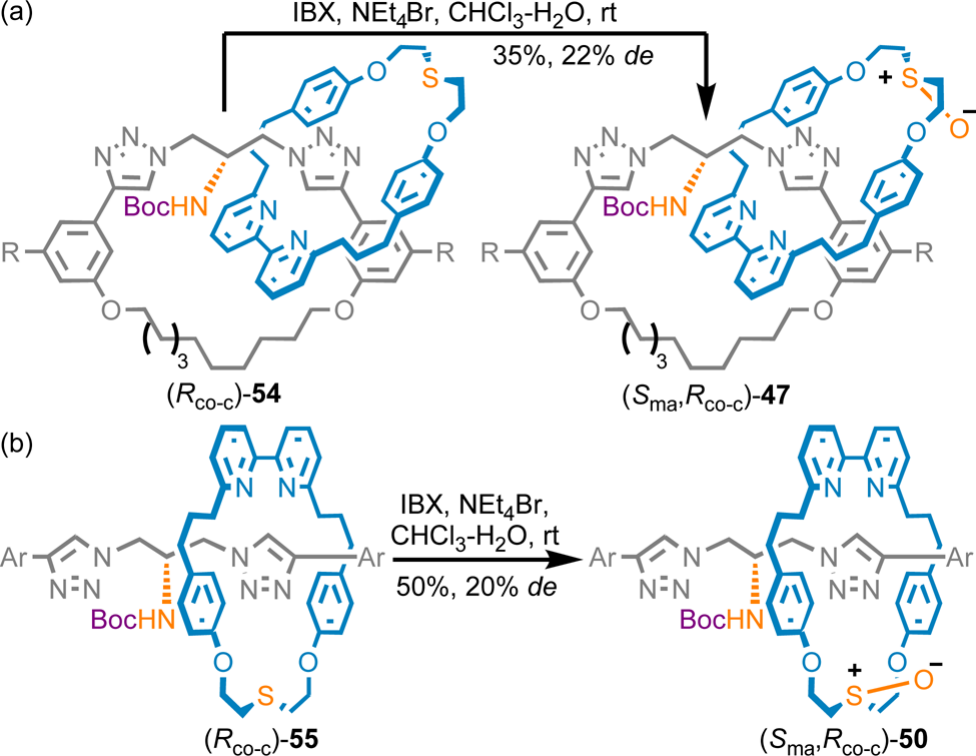
Diastereoselective Oxidation of Co-conformationally Chiral (a) Catenane **54** (R = CO_2_Me) and (b) Rotaxane **55** (Ar = 3,4-di-^*t*^Bu-C_6_H_3_) with 2-Iodoxybenzoic Acid (IBX)^10^

Finally, it is also possible to form the MAC stereogenic unit by taking advantage of co-conformational stereochemistry to control a stereoselective desymmetrization of the faces of one of the components (Scheme 7).^2^ Catenane (*R*_co-c_)-**54**, whose co-conformational configuration is fixed, could be oxidized stereoselectively to give diastereomeric catenanes **47**. Interestingly, the major stereoisomer, (*S*_ma_,*R*_co-c_)-**47**, was the opposite of that obtained in the direct AT-CuAAC synthesis of the same molecule (Scheme 6b). Similarly, rotaxanes **50** could be accessed stereoselectively by oxidation of rotaxane (*R*_co-c_)-**55**, again with opposite stereoselectivity to the direct AT-CuAAC approach (Scheme 6c).

## MGI Catenanes and Rotaxanes (Type 1)

Most MGI-1 rotaxanes^48^ and all reported MGI catenanes^49^ are derived from calixarene or similar^50^ macrocycles, where the facial dissymmetry is provided by a fixed cone-shaped conformation. Systems where the facial dissymmetry is provided by a single prochiral center, as proposed by Schill,^11^ have been largely^51^ overlooked.

**Scheme 8:**
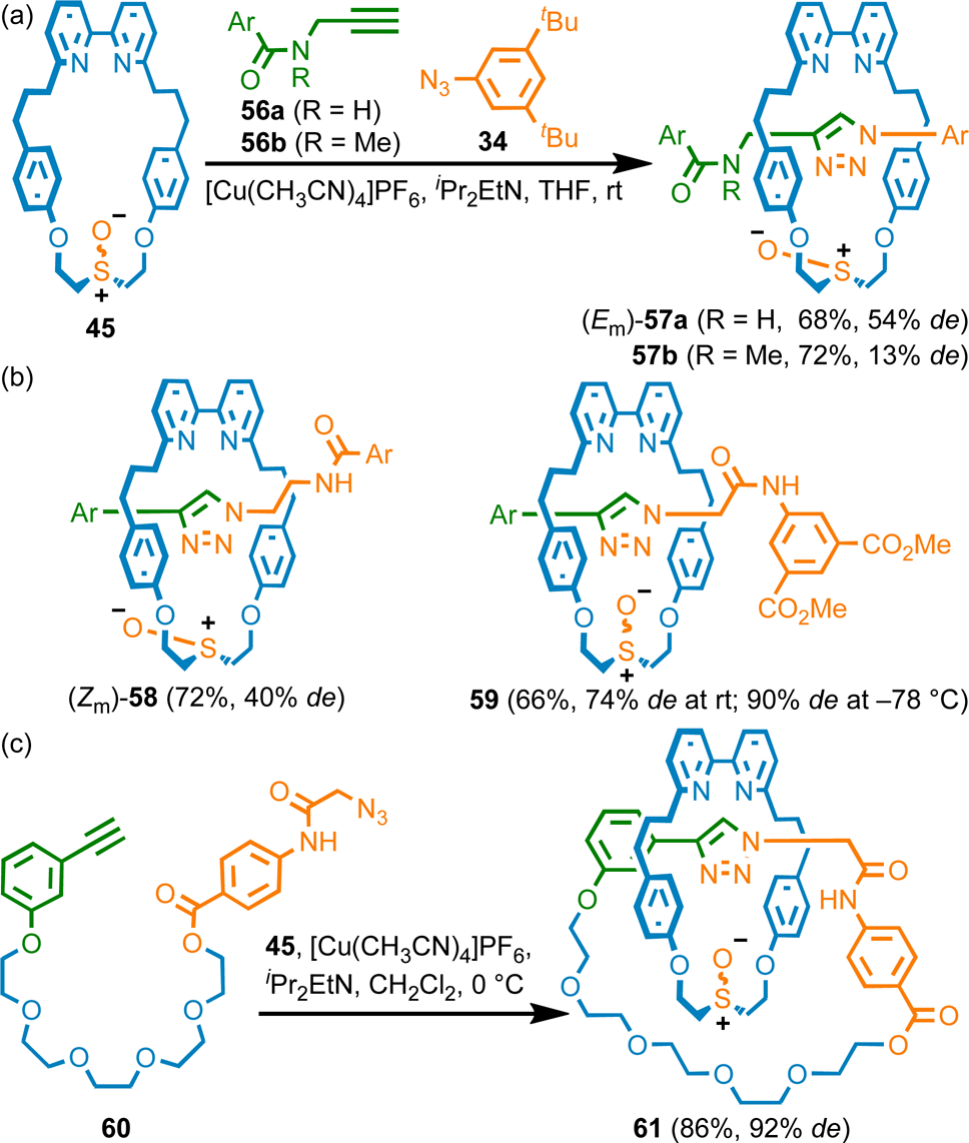
(a) Diastereoselective Synthesis of MGI-1 Rotaxanes **57**. (b) MGI-1 Rotaxanes **58** and **59** Synthesized Stereoselectively from **45**. (c) Diastereoselective Synthesis of MGI Catenane **61**. Ar = 3,4-di-^*t*^Bu-C_6_H_3_^10^

The facial control required for the synthesis of MAC rotaxanes is identical to that required for the synthesis of MGI structures. Thus, we explored the formation of type I MGI rotaxanes using sulfoxide macrocycle **45**. As expected, if one of the coupling partners contained an H-bond donor, a much higher stereoselectivity was obtained; coupling of secondary amide-containing alkyne **56a** gave rotaxane *E*_m_-**57a** (Scheme 8a) in reasonable selectivity (54% *de*). When analogous tertiary amide half-axle **56b** was used, corresponding rotaxane **57b** was produced in low stereoselectivity (13% *de*). Interestingly, rotaxane *Z*_m_-**58**, which is synthesized from a secondary amide containing azide half-axle, was produced stereoselectively (40% *de*) but the macrocycle orientation with respect to the triazole is identical to that of **57a** (Scheme 8b); although the NH···O interaction appears to be important in directing the reaction, the flexibility of macrocycle **45** means that it is hard to predict *a priori* the major isomer formed. Rotaxane **59**, which was designed to contain a more polarized NH unit was formed in high stereoselectivity at rt (72% *de*), which increased to 90% de at −40 °C. Using the same approach (Scheme 8c), catenane **61** could be synthesized from pre-macrocycle **60**, which also contains an electron deficient amide, in good stereoselectivity (92% *de*).

## Synthesis of Type 2 MGI Rotaxanes

**Scheme 9:**
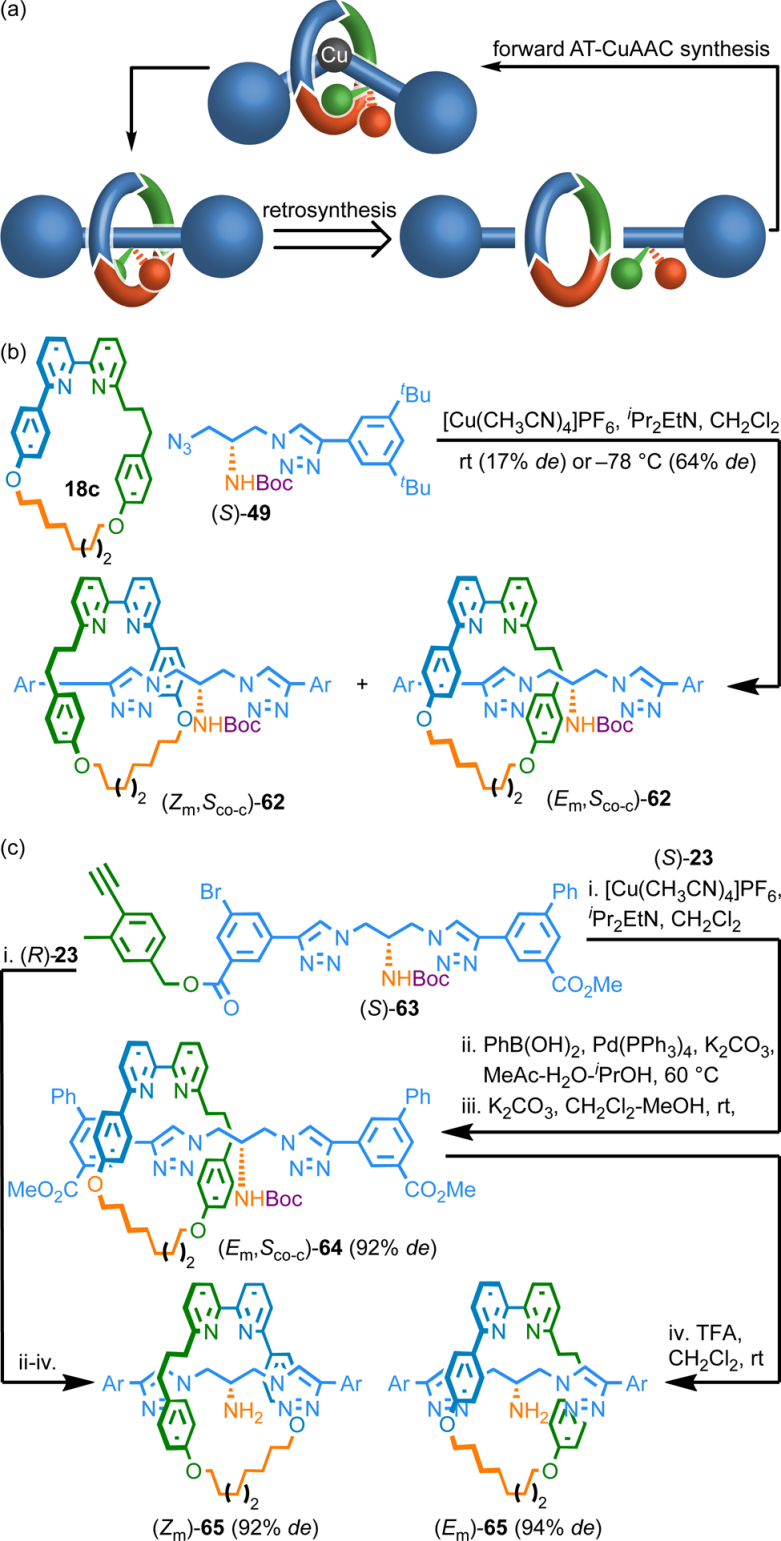
(a) Schematic Retrosynthesis of the MGI-2 Stereogenic Unit. (b) Attempted Direct Diastereoselective Synthesis of Rotaxanes **62** (Ar = 3,4-di-^*t*^Bu-C_6_H_3_). (c) Chiral Interlocking Auxiliary Synthesis of Rotaxanes **65** (Ar = 3-CO_2_Me-5-Ph–C_6_H_3_)^10^

Type 2 MGI rotaxanes present an unusual synthetic challenge. First, as with MAC systems (Scheme 6a), retrosynthetic analysis using a direct AT-CuAAC approach (Scheme 9a) highlights that chiral half-axle synthons are almost inevitable, even though the stereogenic unit itself is nonchirotopic. In the forward synthesis, stereoselectivity could arise through interactions between the substituents of the nascent prochiral unit and the bilaterally dissymmetric macrocycle, which is similar to how selectivity arises in the synthesis of MPC rotaxanes. This analysis also highlights that the same diastereomeric mixture will arise whether the starting materials are racemic or enantiopure.

Unfortunately, the direct AT-CuAAC reaction between macrocycle **18c** and half-axle (*S*)-**49** to give diastereomeric rotaxanes **62**, which contain an MGI-2 stereogenic unit and a co-conformational stereogenic center gave poor selectivity (17% *de*) at rt (Scheme 9b).^21^ This is perhaps unsurprising; the desired process resembles the synthesis of MPC rotaxanes, which we have established requires hindered α-chiral azide auxiliaries,^42^ a problem here as it would necessitate a 1,1 bis-azide building block. Although the selectivity could be improved at the expense of conversion by reducing the reaction temperature, the diastereomers produced were not separable and so we were unable to produce an MGI-2 rotaxane in high stereopurity using this approach.

Thus, we applied our chiral interlocking auxiliary strategy for the synthesis of MPC rotaxanes (Scheme 5), which controls the relative orientation of the axle and macrocycle, to the synthesis of the MGI-2 stereogenic unit (Scheme 9c). Coupling half-axle (*S*)-**63** with α-chiral azide (*S*)-**23** followed by Suzuki coupling and transesterification gave type II MGI rotaxane (*E*_m_,*S*_co-c_)-**64** in 92% *de*.^52^ Removal of the Boc group gave (*E*_m_)-**65** (94% *de*) whose only stereochemistry arises from the MGI-2 stereogenic unit. Repeating the same sequence replacing azide (*S*)-**23** with (*R*)-**23** gave (*Z*_m_)-**65** (92% *de*). It should be noted that stereoselectivity in this synthesis depends on the stereochemical relationship between the azide and the alkyne half-axle components; unlike in the direct synthesis, using racemic coupling partners would result in an equimolar mixture of the product diastereomers.

## Conclusions and Future Directions

**Scheme 10:**
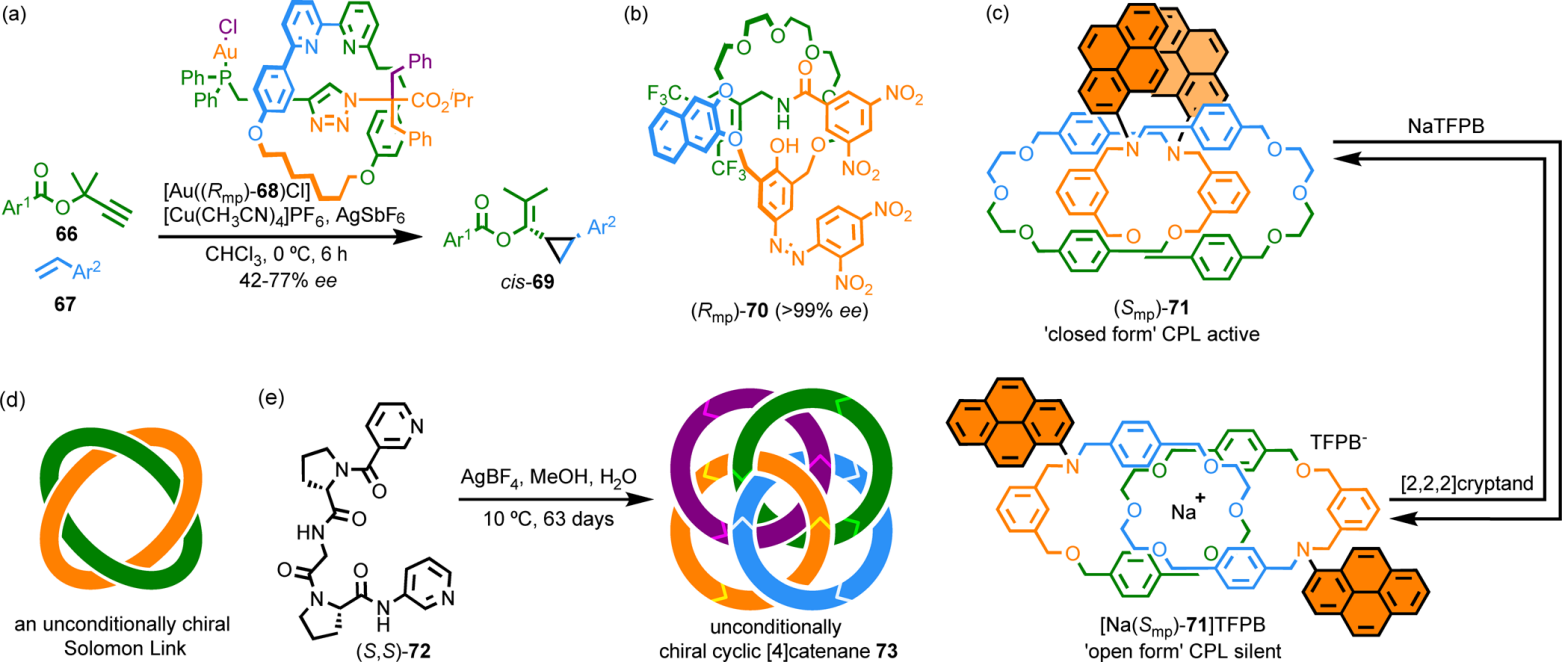
(a) MPC Precatalyst [Au(**68**)Cl]. (b) MPC Rotaxane **70** for the Sensing of Small Chiral Molecules. (c) MPC Catenane **71** that Displays Multistate CPL Switching. (d) Schematic of an Unconditionally Topologically Chiral Solomon Link. (e) Fujita’s Synthesis of Unconditionally Topologically Chiral [4]Catenane **73**(10)

Over the past decade, we have demonstrated that the fundamental mechanical stereogenic units of simple rotaxanes and catenanes can yield to stereoselective synthesis using simple chiral auxiliaries and related approaches. Along the way, we have also identified new mechanical stereogenic units and attempted to systematize the description of mechanically stereogenic molecules, as well as developing self-consistent methods for their assignment. An obvious question of interest to the wider community is what benefits mechanical stereochemistry can bring in chemical applications. We have demonstrated enantioselective catalysis using an MPC rotaxane-based ligand synthesized using our chiral auxiliary strategy (Scheme 10a).^4^ Others have used CSP-HPLC resolution to produce MPC rotaxanes for the sensing of small organic molecules (Hirose; Scheme 10b),^53^ chiroptical switching (Schalley)^54^ and to control the stereochemistry of helical polymers (Takata).^55^ More recently, Yang, Wang, He, and co-workers reported an MPC catenane that exhibits switchable CPL (Scheme 10c).^56^ Based on these prototypical examples, there are clearly opportunities to harness mechanical stereochemistry to solve chemical problems.

If these opportunities are to be realized, further synthetic developments are required. For example, although we have demonstrated general concepts that allow the synthesis of all the fundamental mechanical stereogenic units, we have focused our efforts on the AT-CuAAC reaction because of the synthetic flexibility it brings.^39^ If we are to make functional molecules, we must broaden the structures that are available for study by applying these concepts to other mechanical bond forming reactions. We note that others have developed complementary stereoselective approaches to MPC rotaxanes; Leigh reported a direct enantioselective synthesis in up to 50% *ee* using substrate control,^57^ Kawabata reported a kinetic resolution process that produces an enantiopure product in up to 30% yield^58^ and Tian and Zhu reported a catalytic enantioselective desymmetrization reaction that proceeds in up to 93% *ee*.^59^ The latter examples demonstrate that methods developed for the stereoselective synthesis of covalent structures can be extended to mechanical stereogenic units. Thus, there is clearly an opportunity for those developing stereoselective catalysts to make a significant contribution.

Finally, in tandem with a push toward understanding the benefits mechanical stereochemistry may bring and broadening the generality of the approaches demonstrated so far, it is worth considering where the next stereochemical horizons lie. It is hopefully now clear that synthetic progress goes hand in hand with a proper characterization of stereogenic units; until recently, the structural complexity of even two component interlocked molecules obscured the underlying nature of the stereogenic units that can arise. As the number of crossing points increases, as exemplified by Solomon links (Scheme 10d),^60^ or the number of interlocked components increases, as in the case of cyclic [n]catenanes (Scheme 10e),^61^ other opportunities for mechanical stereochemistry arise. Although examples of such systems have been synthesized stereoselectively through the assembly of covalent chiral components, no examples of these structures where the mechanical bond provides the sole source of stereoisomerism have been reported, a similar situation to that which pertained in simple systems when we began our work. Also similarly, the fundamental nature of these stereogenic units is often unclear.

The understanding gained during the studies described in this Account put us in a strong position to attack these next challenges, as well as demonstrating the value in systematizing the discussion of mechanical stereochemistry beyond simple [2]catenanes and rotaxanes. It is our contention that, having established clear definitions of the fundamental mechanical stereogenic units and demonstrated that they can yield to simple synthetic concepts, we have finally reached the end of the beginning, 63 years after Wasserman and Frisch highlighted the potential for mechanical stereochemistry to arise in catenanes—the golden age of mechanical stereochemistry lies ahead!

## Supporting Information Available

Instructions for the assignment of mechanical stereogenic units (PDF)

## Dedication

Dedicated to Professor David Leigh in the year of his 60th birthday.
