# Supplementary material for: The End of the Beginning of Mechanical Stereochemistry
Source: Acc Chem Res. 2024 Jun 3;57(12):1696–708. doi: 10.1021/acs.accounts.4c00195 (PMC11191403; doi:10.1021/acs.accounts.4c00195)
Supplement: Supplementary file 1 — ar4c00195_si_001.pdf [file ar4c00195_si_001.pdf]

Electronic Supporting Information

The End of the Beginning of Mechanical Stereochemistry

Stephen M. Goldup*

School of Chemistry, University of Birmingham, University Rd W, Birmingham B15 2TT
*s.m.goldup@bham.ac.uk

1 Absolute stereochemical assignment of interlocked compounds – our approach 3

2 Absolute stereochemical assignment of interlocked compounds – general considerations 3

3 Assigning the mechanically planar chiral stereogenic unit – method and worked examples 5

3.1 Step by step approach 5

3.2 Worked example – MPC catenane 1 5

3.3 Worked example – MPC rotaxane 3 6

4 Mechanically axially chiral catenanes and rotaxanes 7

4.1 Step by step approach 7

4.2 Worked example – MAC catenane 2 7

4.3 Worked example – MAC rotaxane 51 8

5 Mechanical geometric isomers 9

5.1 Worked example – MGI catenane 61 9

5.2 Worked example – MGI-1 rotaxane 57a 10

5.3 Worked example – MGI-2 rotaxane 65 11

6 Determining orientation and facial dissymetry in systems containing covalent stereochemistry 12

6.1 Worked example – peptidic catenane 7 12

6.2 Determining orientation and facial dissymmetry in cyclodextrins (CDs) 13

6.3 Worked example – catenane 6 14

7 Co-conformational stereoisomers 14

8 “Conformational” Mechanical Stereochemistry 15

8.1 Worked example – rotaxane 4 (method 1 – used in the manuscript) 16

8.2 Worked example – rotaxane 4 (method 2) 16

9 References 17

1 Absolute stereochemical assignment of interlocked compounds – our approach

We proposed methods for the assignment of absolute stereochemistry in mechanically planar chiral molecules based on two oriented components, and mechanically axially chiral molecules based on facially dissymmetric components in an early review.[1] As the number of mechanical stereogenic units has expanded, these methods have been developed. As discussed in the manuscript, our guiding principles when developing these methods, in addition to honoring any previous proposals where possible, have been:

i. As the MAC, MPC and MGI rotaxane and catenane pairs are related by a notional ring opening process, the method used to assign them should avoid automatic inversion of the stereolabel when this process is considered;

ii. Globally, the methods developed should require the minimum number of arbitrary rules. Most obviously, the orientation of the components and the observer when the vectors are viewed should be the same throughout.

These methods, which are outlined in subsequent sections are only proposals – none have been adopted officially. Furthermore, some have already been subjected to revision. For example, our original method[2] to assign mechanically axially chiral (MAC) catenanes was based on a proposal from Stoddart and Bruns.[3] We then used condition (i) to derive the method for the corresponding MAC rotaxanes, which importantly require the relative orientation of macrocycle and axle to be specified. However, we subsequently found that the method for assigning MAC rotaxanes could not be extended properly MGI-2 rotaxanes without failing test (i) or (ii); if the same relative orientation of axle and macrocycle as used in MAC rotaxanes was applied in MGI-2 rotaxanes, the stereolabel inverted compared with the corresponding MGI catenane.[4,5] Similarly, although we and others originally proposed a "steering wheel" method for assigning the MPC stereogenic unit, we have now settled on an oriented skew line approach, as this is then consistent between MAC, MPC and MGI stereochemistry. Thankfully, the steering wheel and skew line approaches give the same stereolabel and so this requires no revision of previous assignments.

2 Absolute stereochemical assignment of interlocked compounds – general considerations

Our current methods for the assignment of mechanical stereogenic units all make use of Cahn-Ingold-Prelog (CIP)-derived atom priorities to unambiguously assign vectors associated with bilateral dissymmetry of the covalent sub-components. The stereochemical assignment is then achieved by considering the relative orientation of the vectors associated with the individual components (Figure S1). Once the vectors are established, a key consideration is the position at which the vectors are observed; viewing the vectors associated with the MAC and MGI stereogenic units at different positions or with the axle component at different relative orientations with respect to the observer in the case of rotaxanes can yield different results.

i. The (pro)stereogenic center of the macrocycle (MGI, MAC systems) should be arranged such that the in-macrocycle plane substituents point towards the center of the ring.

ii. The (pro)stereogenic center of the rotaxane axle (MGI-2, MAC) should be arranged with the in-axle substituents pointing towards the observer.

iii. Catenane vectors should be observed from a position within one of the macrocycles and towards the other. Rotaxanes vectors are observed from the macrocycle towards the axle.

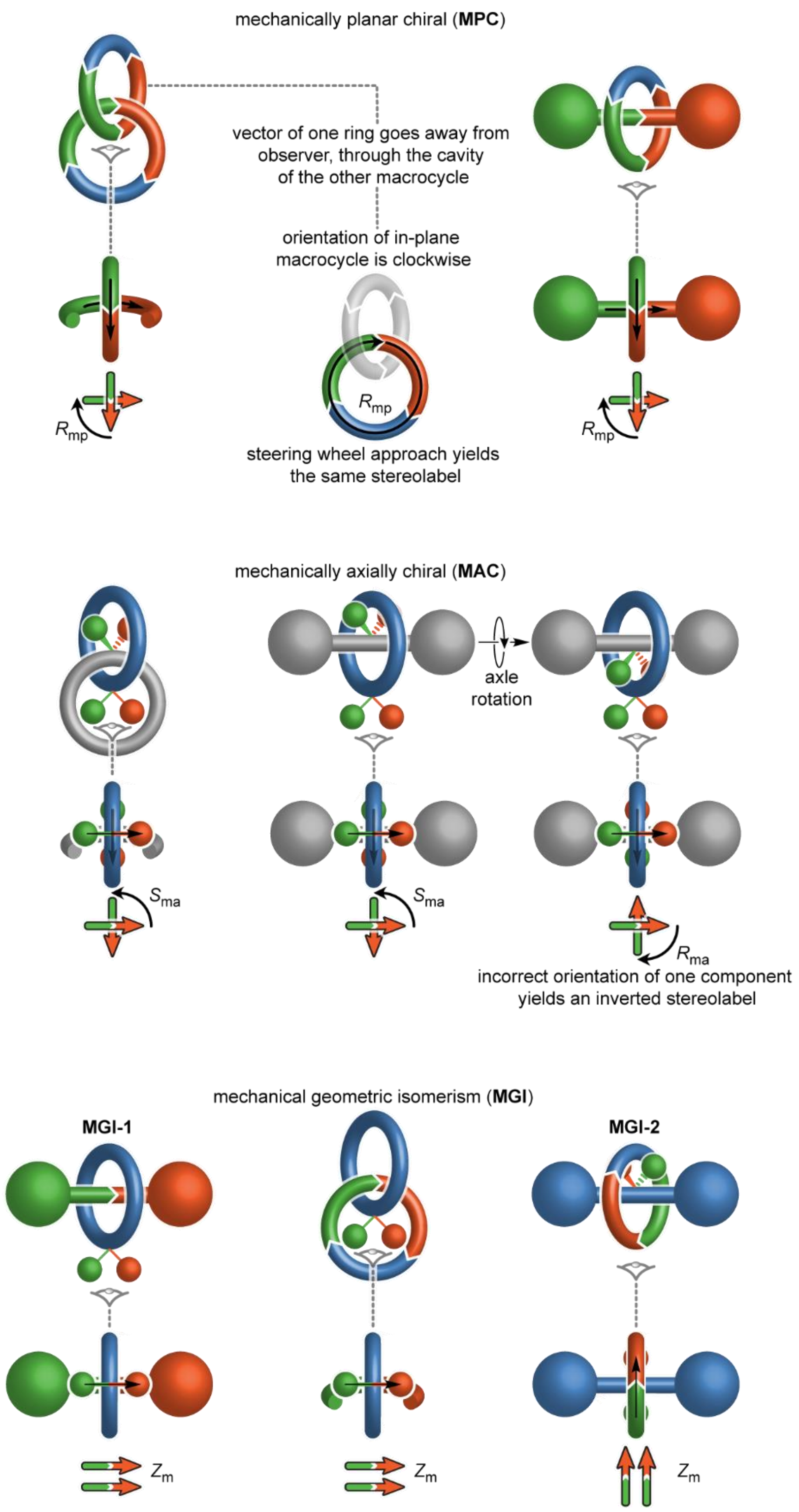

Figure S1. Schematic representation of the simple mechanical stereogenic units, the correct orientation of the components when assigning stereochemistry and the view taken when observing the vectors.

As noted above, the skew line approach for the assignment of MPC systems replaces our original 'steering-wheel' approach. By considering that the plane of the macrocycle is being desymmetrised by the other macrocycle, the head of the vector A → B vector of the axle (rotaxanes) or ring for (catenanes) passing through the cavity of the other ring away from the observer, can be considered as the 'pilot atom' in the analogous classical planar chiral stereogenic unit. The orientation of the ring parallel to the plane of the observer can then be used to define the stereolabel: clockwise = R_{mp}, counterclockwise = S_{mp}. This gives the same assignment as the skew lines.

3 Assigning the mechanically planar chiral stereogenic unit – method and worked examples

3.1 Step by step approach

1) Following to the Cahn-Ingold-Prelog (CIP) rules, identify the highest priority atom on one ring and label it as "**A**"

2) Moving outward from A in spheres, as per the CIP method for assigning covalent stereogenic centres, determine the highest priority atom (CIP) that can be used to define an orientation of the ring (typically a ligand of **A**) and label it as "**B**". If A does not lie within the ring structure, the same method is used but atom **B** is the atom in the earliest sphere that allows direction to be defined. The orientation of the ring is defined by the vector A → B, which, where relevant, passes through the intervening atoms (*i.e.*, follows the bonds).

3,4) Repeat steps (1) and (2) on the second subcomponent to identity its orientation.

5) Reduce the assembly to the corresponding vectors and observe their relative orientation at the crossing point within the rings (catenanes) or from the macrocycle to the axle (rotaxanes).

6) If the path from the head of the front vector to the tail of the rear vector corresponds to a clockwise path the stereolabel is assigned as R_{mp}, counterclockwise = S_{mp}. The "mp" subscript is included to indicate that the stereodescriptor refers to a mechanically planar stereogenic unit.

3.2 Worked example – MPC catenane 1

Step 1: The atom of the macrocycle with the highest priority is one of the polyethylene-glycol chain oxygen atoms immediately bonded to a phenyl ring. The earliest atom of difference between **A** and **A`** is the 4/7-position in the phenanthroline unit. As the center encountered by **A** bears a phenyl substituent, this is the highest CIP priority.

Step 2: The highest priority ligand of **A** is the carbon of the aromatic ring, which allows the orientation of the macrocycle to be assigned, and so is labelled **B**.

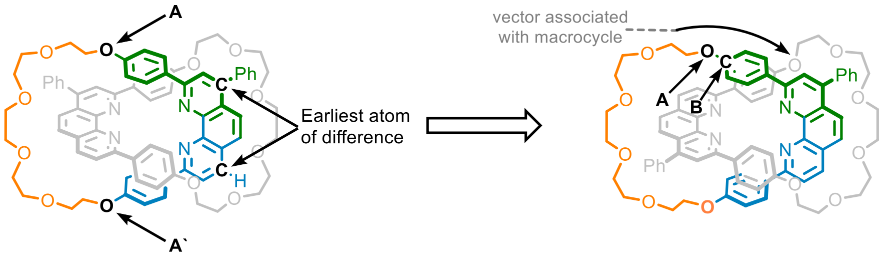

Step 3 and 4: As the rings are identical, the same atoms used to define the orientation of the second macrocycle.

Step 5: Reduce the structure to the corresponding vectors and observe their orientation at the crossing point between the rings.

Step 6: As a clockwise path is taken from the head of the front vector to the tail of the rear vector the stereoisomer of catenane **1** shown is labelled as (S_{mp})-**1**.

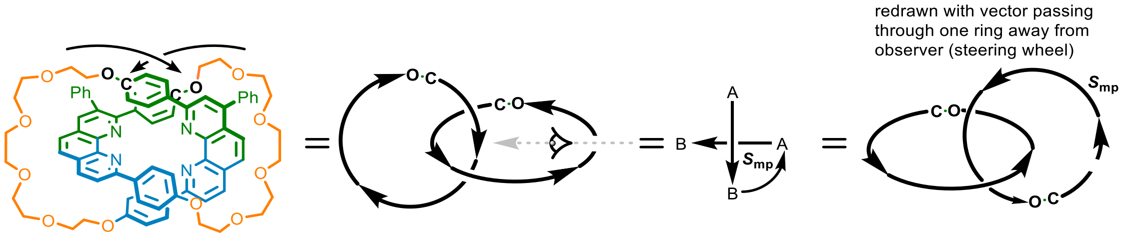

3.3 Worked example – MPC rotaxane 3

Step 1: The atom of the macrocycle with the highest priority is the S, which is labelled **A**.

Step 2: The highest priority ligand of **A** that allows direction to be assigned (note: the exocyclic O ligands are not useful here) is the sulfonamide N, which is labelled **B**.

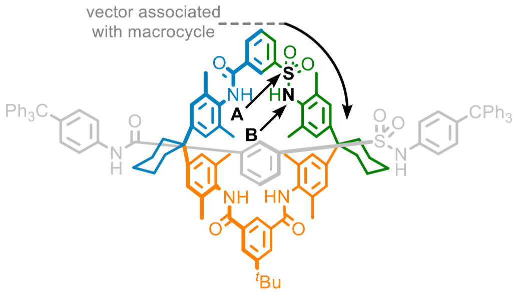

Step 3: The atom of the axle with the highest priority is the S, which is labelled **A**.

Step 4: The highest priority ligand of **A** the allows direction to be assigned (note: the oxygen ligands of S are not used as they do not provide direction) is the sulfonamide N, which is labelled **B**.

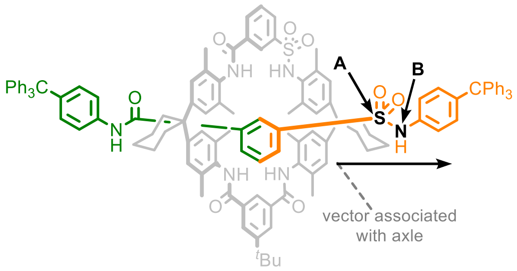

Step 5: Reduce the assembly to a line structure bearing the corresponding vectors and observe their relative orientation at the crossing point from the macrocycle to the axle.

Step 6: As an anticlockwise path is taken to the head of the vector defined by the macrocycle (front) to the tail of the vector defined by the axle (rear), the stereoisomer of rotaxane **3** shown is labelled as (S_{mp})-**3.**

Note: The "steering wheel" approach (also shown) gives the same stereolabel.

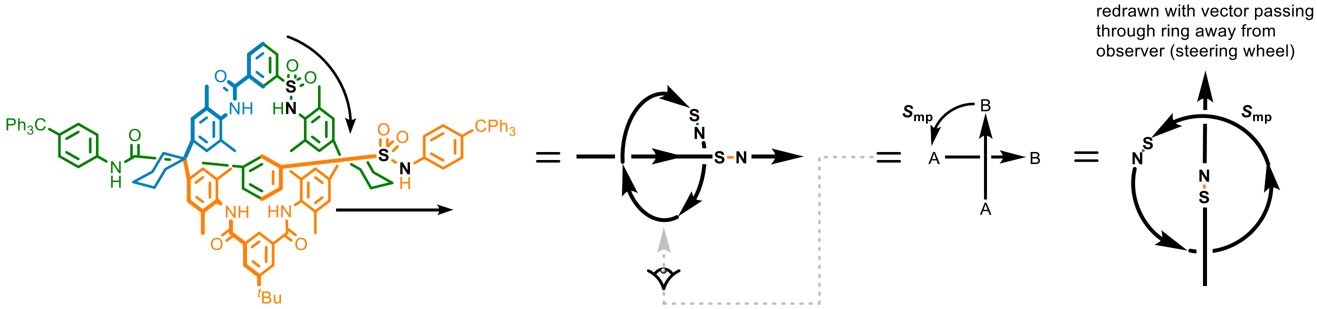

4 Mechanically axially chiral catenanes and rotaxanes

4.1 Step by step approach

Step 1: Identify the highest priority prochiral group (or stereogenic unit in *meso* structures) in the macrocycle using the Cahn-Ingold-Prelog priority of the central atom. Redraw the ring such that the substituents of the prochiral in the plane of the macrocycle point into its center.

Step 2: Identify the highest priority ligand of the selected center that lies outside of the macrocycle plane, again using CIP priority, and label it as "**A**"; label the lower priority group "**B**".

Steps 3 & 4: Repeat steps 1 and 2 for the second component. If the second component is an axle (i.e. a rotaxane), orient the selected center such that in-axle substituents are projected towards the observer.

Step 5: Reduce the assembly to the corresponding vectors and observe their relative orientation at the crossing point within the rings (catenanes) or from the macrocycle to the axle (rotaxanes).

Step 6: If the path from the head of the front vector to the tail of the rear vector corresponds to a clockwise path the stereolabel is assigned as R_{ma}, counterclockwise = S_{ma}. The "ma" subscript is included to indicate that the stereodescriptor refers to a mechanically planar stereogenic unit.

4.2 Worked example – MAC catenane 2

Step 1: The highest CIP prochiral center is the tertiary carbon bearing the phenyl and proton substituents. The prochiral center is already drawn in the correct orientation with respect to the ring (in plane substituents point into the cavity).

Step 2: The highest priority substituent of this prochiral center is the C atom, which is labelled as "**A**", and so the H atom is labelled "**B**".

Steps 3 and 4: The rings are identical and so the same atoms are used to define the faces of the second macrocycle.

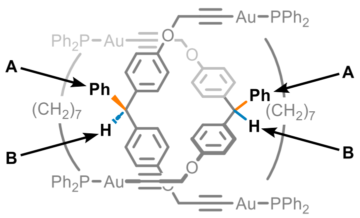

Step 5: Reduce **2** to a line structure with each vector embedded in the cavity of the other ring.

Step 6: As a clockwise path is taken from the head of the front vector to the tail of the rear vector the stereoisomer of catenane **1** shown is labelled as (R_{ma})-**2.**

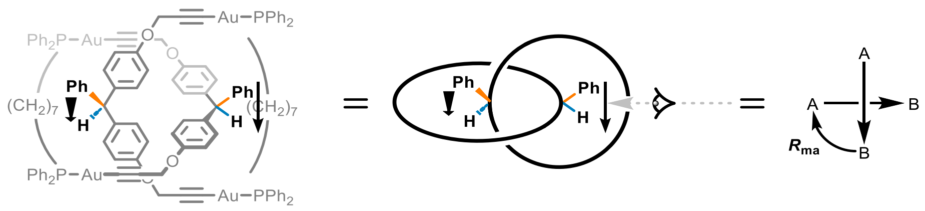

4.3 Worked example – MAC rotaxane 51

Step 1: The highest CIP prochiral center in the macrocycle is the sulfoxide unit, which is redrawn such that the in-plane methylene units point into the cavity of the ring.

Step 2: The highest priority exocyclic atom bonded to the sulfoxide according to CIP rules is the oxygen, which is labelled as "**A**", while the lone pair is labelled as "**B**".

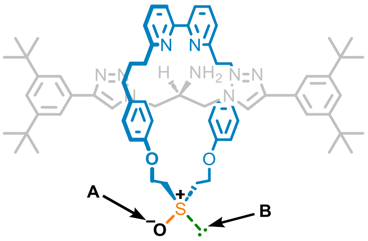

Step 3: The highest priority prochiral center in the axle is the carbon bearing the amine unit. This is redrawn such that the in-axle substituents are oriented towards the observer (looking up in plane of the page, as indicated).

Step 4: The highest priority exocyclic atom bonded to the prochiral carbon is the amine nitrogen, which is labelled as "**A**". The proton is labelled as "**B**".

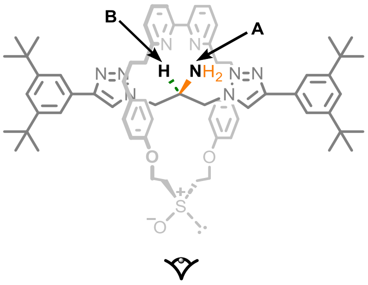

Step 5: Reduce the structure to a line drawing bearing the assigned vectors, taking care not to reorient the axle and arranging the components so that the macrocycle vector is encountered first by the observer.

Step 6. As a clockwise path is taken from the head of the front (macrocycle) vector to the tail of the rear (axle) vector, the stereoisomer shown is labelled (S_{ma})-**51**.

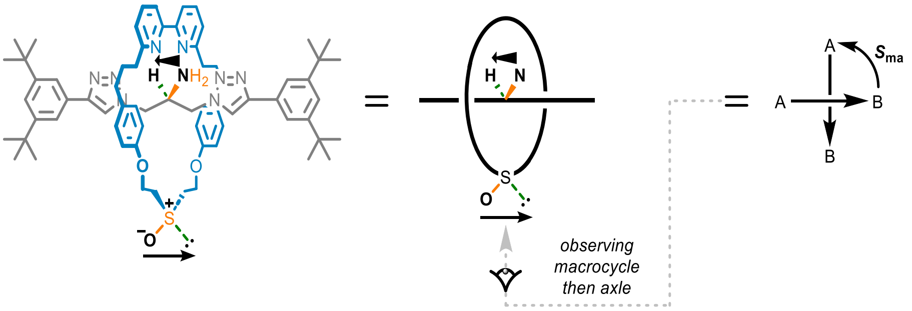

5 Mechanical geometric isomers

The method of assignment for the mechanical geometric isomers is derived from the methods used to assign the MAC and MPC stereogenic units; the same approach is used to determine the orientation/facial dissymmetry and the same relative orientation of axle and macrocycle (rotaxanes) is used. The key difference is that the vectors obtained can be arranged co-planar either syn- or anti-periplanar. Thus, for each of the geometric stereogenic units, one simply derives the vectors associated with each component as above and then views them in the require orientation, again, as described above. If the vectors are syn to one another, the stereogenic unit is defined as Z_m whereas anti gives rise to E_m.

5.1 Worked example – MGI catenane 61

Step 1: The highest priority atom in the oriented macrocycle is the alkyl O of the ester, which is labelled as "**A**".

Step 2: The highest priority ligand of **A** is the carbonyl C, which is labelled as "**B**".

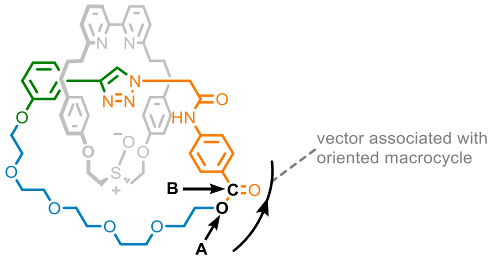

Step 3: The highest priority prochiral center in the macrocycle is the sulfoxide unit, which is redrawn such that the in-plane methylene units point into the cavity of the ring.

Step 4: The highest priority out of plane atom is the O, which is labelled as "**A**". The lone pair is labelled as "**B**".

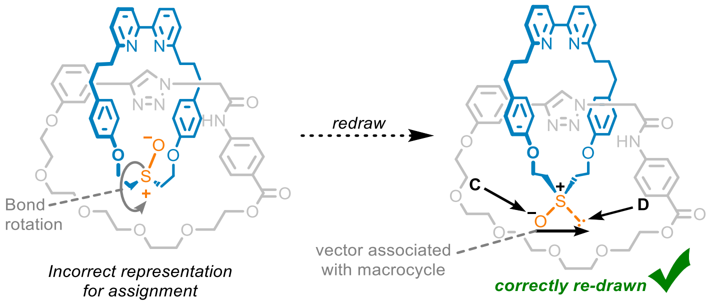

Step 5: Reduce the assembly to the respective vectors.

Step 6: View the relative orientations of the vectors at the crossing point between the rings. As they point in opposite directions, the stereoisomer shown is (E_m)-**61.**

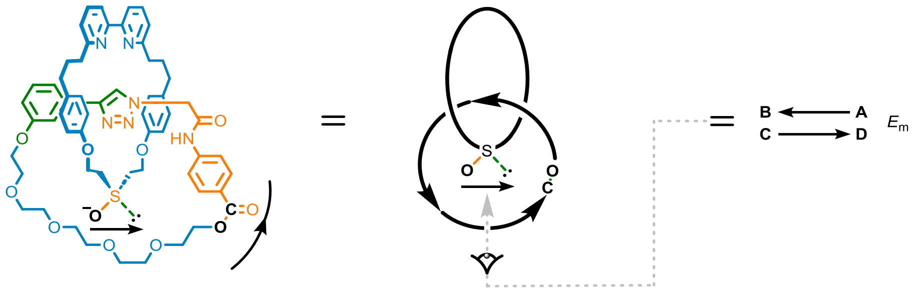

5.2 Worked example – MGI-1 rotaxane 57a

Step 1: The atom of the axle with the highest priority is the amide O. This is labelled as **A.**

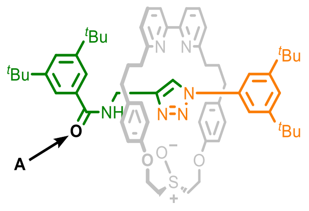

Step 2: Moving outward from **A**, the highest priority atom which allows the orientation of the axle to be assigned is amide N. This is labelled **B**.

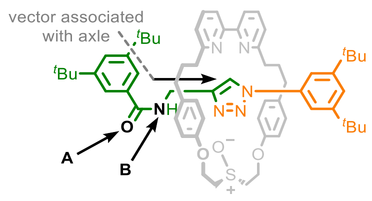

Step 3: The highest priority prochiral center in the macrocycle is the sulfoxide unit, which is redrawn such that the in-plane methylene units point into the cavity of the ring.

Step 4: The highest priority out of plane atom is the O, which is labelled as "**A**". The lone pair is labelled as "**B**".

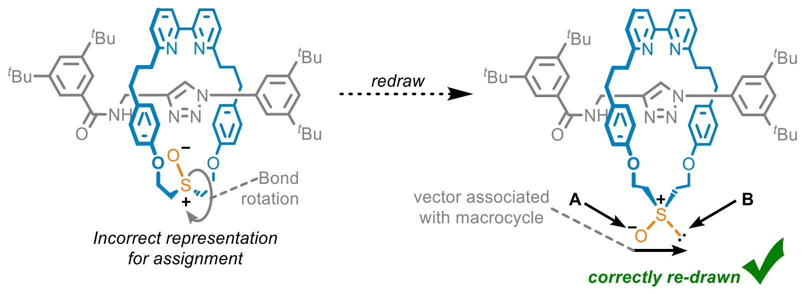

Step 5: The vectors **A**→**B** (associated with the axle) and **A**→**B** (associated with the macrocycle) point in the same direction and so the stereoisomer shown is (Z_m)-**57a**.

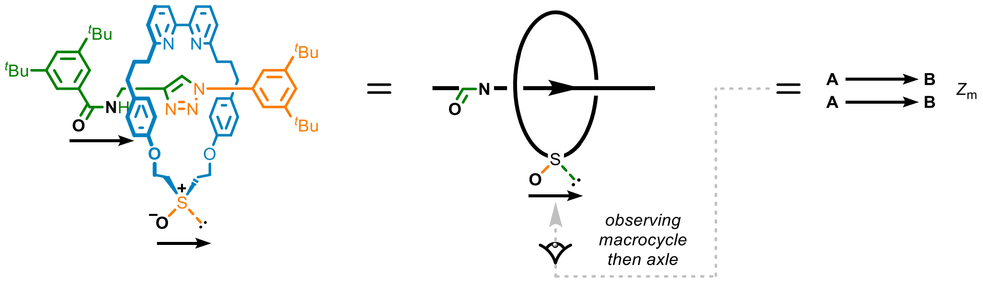

5.3 Worked example – MGI-2 rotaxane 65

Step 1: The highest priority in the macrocycle is the phenolic ether O highlighted. This is labelled **A**.

Step 2: The highest priority ligand of **A** is the quaternary C of the aromatic ring. This is labelled **B.**

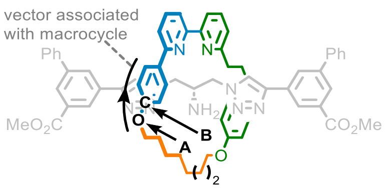

Step 3: The highest priority prochiral center in the axle is the carbon bearing the amine unit. This is redrawn such that the in-axle substituents are oriented towards the observer.

Step 4: The highest priority exocyclic atom is the amine N. This is labelled "**A**". The proton is labelled "**B**".

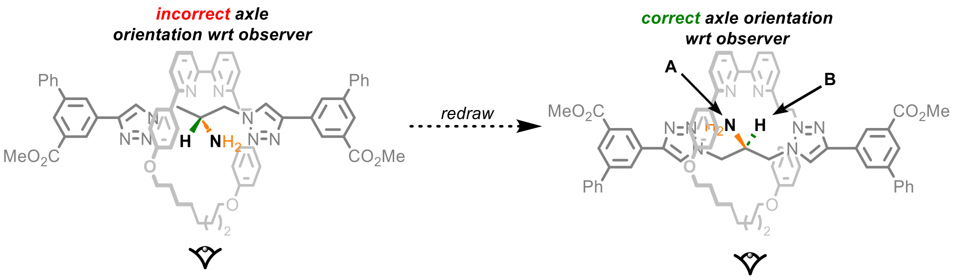

Step 5: Reduce the assembly to the respective vectors.

Step 6: View the relative orientations of the vectors. As they point in opposite directions, the stereoisomer shown is (E_m)-**65.**

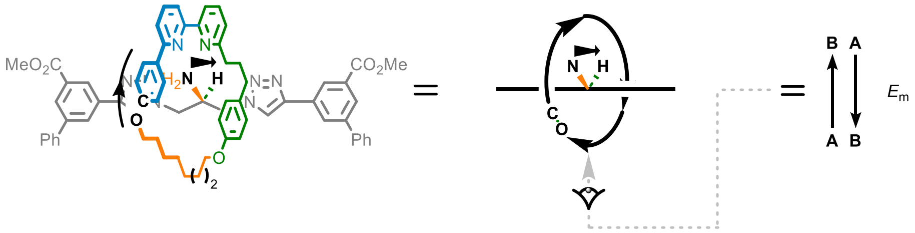

6 Determining orientation and facial dissymetry in systems containing covalent stereochemistry

As discussed in the manuscript, if a macrocycle or axle contains a covalent stereogenic centre embedded such that it both orients and provides facial dissymmetry, a molecule expresses both covalent and mechanical stereochemistry and the stereochemistry can be described using two of three possible stereodescriptors. Assigning the vectors associated with the orientation and facial dissymmetry can be achieved exactly as described above for the MPC (identify the highest priority atom [**A**] and the associated highest priority ligand [**B**] that defines the orientation) and MAC (identify the highest priority stereogenic unit; assign the ligands that lie outside the macrocycle plane **A** and **B** in order of priority). The associated stereolabels are then assigned exactly as above, with the caveat that, given that there are always two perpendicular vectors associated with the component containing the stereogenic center, more than one mechanical stereolabel can be assigned.

6.1 Worked example – peptidic catenane 7

Assigning the orientation of a ring:

Step 1: The highest priority atom is the carbonyl O of the phenylalanine hydrazide. This is labelled **A**.

Step 2: The highest priority ligand of **A** that defines the orientation of the macrocycle is the N. This is labelled **B**.

Assigning the facial dissymmetry of a ring:

Step 1`: The highest priority stereogenic center is the α carbon of the phenylalanine unit.

Step 2`**: The highest priority exocyclic atom is the C of the Bn unit. This is labelled "**A`". The H is labelled as "**B`**".

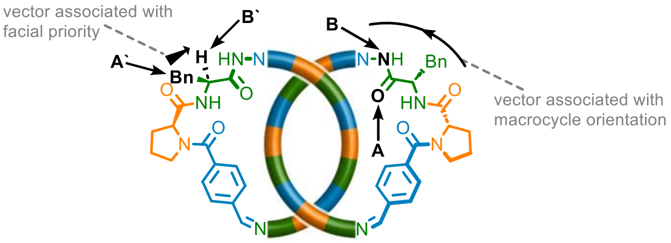

Steps 3/3` and 4/4`: As the rings are identical, the same atoms used to define the orientation and facial dissymmetry of the second macrocycle.

Step 5: Reduce the structure to the corresponding vectors and observe their orientation at the crossing point between the rings.

Step 6: Viewing the oriented vectors, an anti-clockwise path is taken from the head of the front vector to the tail of the rear vector and so an S_{mp} label is applied. Alternatively, viewing the facial priority vectors, a clockwise path is taken from the head of the front vector to the tail of the rear vector and so an R_{ma} label is appropriate. Thus, the molecule can be assigned as $(S,S)_8$-(S_{mp})-**7** or $(S,S)_8$-(R_{ma})-**7**.

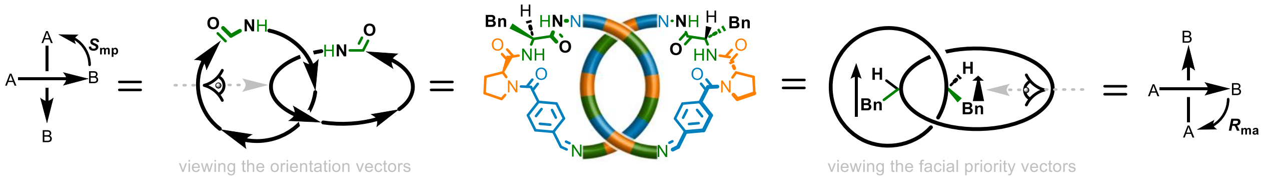

6.2 Determining orientation and facial dissymmetry in cyclodextrins (CDs)

Cyclodextrin rings contain covalent stereogenic centers that give rise to both orientation and facial dissymmetry. They are relatively commonly used in the synthesis of interlocked molecules but the stereochemistry of their products is often poorly described and so worthy of further comment here. Depictions of cyclodextrins typically focus on their cone-shaped conformation and, perhaps as a result, the vast majority of the discussion of the stereochemistry of MIMs they contain has largely focussed on the associated potential for MGI stereochemistry. However, to our knowledge, no attempt has been made to define the facial dissymmetry of these rings, and typically their oriented nature is overlooked.

Defining the orientation of a CD ring is trivial using the rules described above:

Step 1: The highest CIP atom in the glucopyranose repeating unit is the bridging oxygen atom. This is labelled **A**.

Step 2: Moving outward from **A** in spheres, the highest priority ligand is the anomeric C. This is labelled **B**.

Thus, the fixed orientation of the cyclodextrin ring is as shown below.

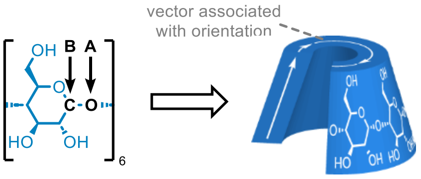

The vector associated with the facial dissymmetry of CD is determined using the same strategy as for macrocycles containing prochiral centers:

Step 1: The highest priority stereogenic center in the ring is the anomeric carbon. The ring should then be redrawn to place the in-plane substituents (the two Os) pointing into the center of the ring.

Step 2: The highest priority exocyclic ligand of the anomeric position is the C atom (labelled **A**), the lowest priority is the H (labelled **B**).

Thus, the facial dissymmetry of the cyclodextrin ring (note this does not depend on its conformation – it is a fixed property of the structure) is as shown below.

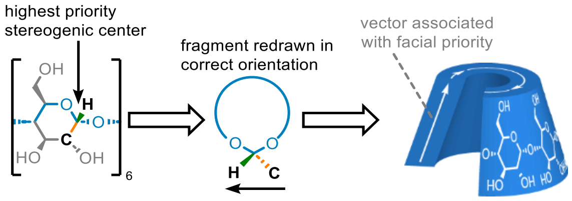

6.3 Worked example – catenane 6

Step 1: The highest priority atom in the biphenyl ring is the carbonyl O of the amide bearing the longer ethylene glycol chain, which is labelled "**A**".

Step 2: Moving outwards from **A**, the highest priority atom that allows the orientation of the ring to be assigned is the N of this amide.

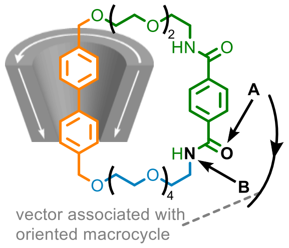

From here, and using the vectors associated the CD ring defined above, we can assign both a MAC and MPC stereolabel to catenane **6** using the methods described previously. The full structure is assigned using one of these mechanical stereolabels combined with the required covalent stereolabels, which can be conveniently represented as D (the cyclodextrin shown is derived from D-glucose). Thus, the isomer shown ca be labelled as (D,E_m)-6 or (D,R_{mp})-**6**, of which we prefer the latter as it highlights the potential for topological stereochemistry, as is indeed the case here.

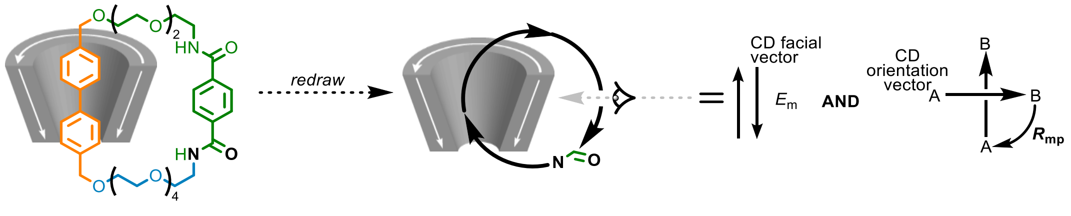

(D,E_m)-**6** OR (D,R_{mp})-**6**

7 Co-conformational stereoisomers

Co-conformational mechanical stereochemistry arises when the position of one component desymmetrizes the other. This can be handled by considering the interlocked component as "ghost" substituent of the component it desymmetrises. The hardest problem that can arise in such systems is determining which section of the desymmetrised component the macrocycle should be considered as encircling – in principle it is possible for this to be ambiguous. However, in all examples reported to date, this is clear. Of course, co-conformational stereochemistry is potentially dynamic in nature and so the co-conformation (or family of co-conformations – i.e. all those where the macrocycle is displaced to the same side of the desymmetrised component) must be specified. Example from the manuscript with notes on their assignment are shown in Figure S2.

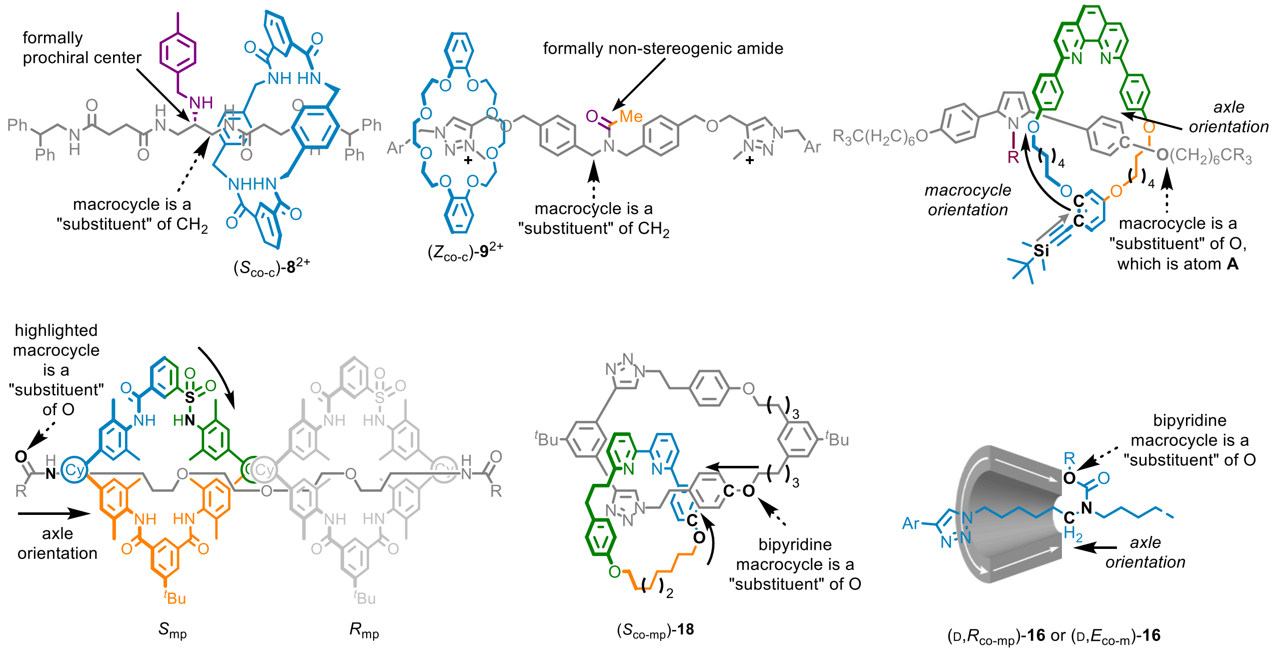

Figure S2. Examples of co-conformationally stereogenic MIMs from the manuscript with notes on their assignment.

8 “Conformational” Mechanical Stereochemistry

Conformational stereochemistry arises when an otherwise non-stereogenic substructure adopts a stereogenic conformation, the most well-known example being rotation about the C-C bond of a biaryl unit. Such conformations can be dynamic or static on the timescale of a measurement, depending on the steric hindrance to conformational exchange. Such conformational motion can also, in principle, give rise to the appearance of mechanical stereogenic units if the molecule adopts a conformation that results in a defined orientation or facial dissymmetry. Indeed, most covalent subcomponents will adopt such conformations but given that these fluxional conformational mechanical stereogenic units are rapidly exchanging, the influence is negligible.

The only obvious current exceptions to this are rotaxanes and catenanes composed of rings that adopt a single, atropisomeric conformation, specifically calixarenes and potentially pilarenes. These can both be synthesized in ring-differentiated forms and, once threaded by an axle or another macrocycle, the exchange between conformations is prevented/severely hindered. However, despite being ubiquitous in the chemistry of rotaxanes and catenanes, to our knowledge, methods to assign the priority of the faces have not been reported.

Unfortunately, the facial priority of these rings cannot be assigned directly using the approach described above for systems bearing a (pro)stereogenic center. For completeness, we suggest two possible methods below using rotaxane **4** as an example. We note that, although both seem reasonable, the result in opposite labels for rotaxane **4**, highlighting both the arbitrary nature of stereolabels (they both define the same structure) and the need to agree common rules to enable accurate discussions.

8.1 Worked example – rotaxane 4 (method 1 – used in the manuscript)

Step 1: The highest priority exocyclic atom bonded to the ring is the urea oxygen. This is labelled **A**.

Step 2: Working outward from A in spheres, the highest priority atom that allows a facial vector to be defined is the indicated N atom. This is labelled **B**.

Steps 3&4: assign the orientation of the axle using the methods described above.

Step 5: The vectors associated with the axle and the macrocycle point in the opposing directions and so the stereoisomer of rotaxane **4** shown is labelled as (E_m)-**4**.

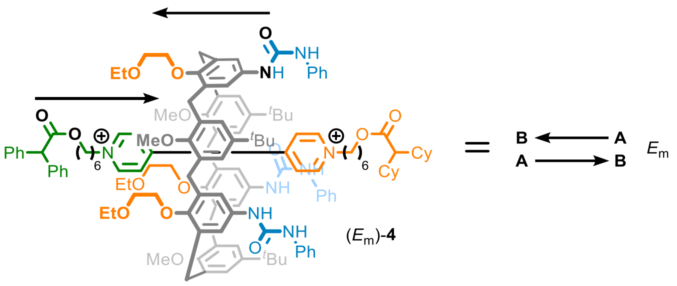

8.2 Worked example – rotaxane 4 (method 2)

Step 1: Define the macrocycle plane by tracing the shortest continuous path around the ring (highlighted in black). If two paths have the same length, the path that contains the highest priority atom takes precedence.

Step 2: identify the highest priority atom directly bonded to an atom in the path defined in step 1 that lies off the macrocycle plane in the conformation of interest. Here this is the phenolic ether O. This is labelled **A**. The atom to which it is bonded is labelled **B**. The facial priority of the ring (i.e. the vector) is defined from the **A** to **B**.

Steps 3&4: assign the orientation of the axle using the methods described above.

Step 5: The vectors associated with the axle and the macrocycle point in the same directions and so the stereoisomer of rotaxane **4** shown is labelled as (Z_m)-**4**.

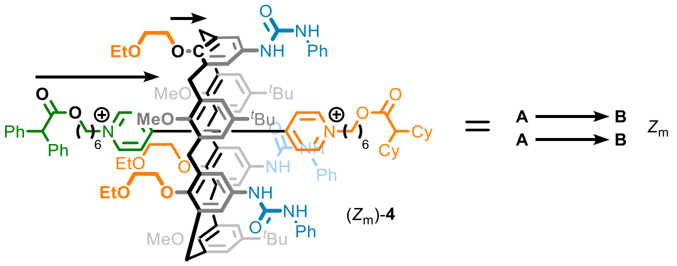

9 References

1. Jamieson, E. M. G.; Modicom, F.; Goldup, S. M., Chirality in rotaxanes and catenanes. *Chem. Soc. Rev.* **2018**, *47* (14), 5266.

2. Maynard, J. R. J.; Gallagher, P.; Lozano, D.; Butler, P.; Goldup, S. M., Mechanically axially chiral catenanes and noncanonical mechanically axially chiral rotaxanes. *Nat. Chem.* **2022**, *14* (9), 1038.

3. Bruns, C. J.; Stoddart, J. F., *The Nature of the Mechanical Bond: From Molecules to Machines*. Wiley: 2016.

4. Gallagher, P. R.; Savoini, A.; Saady, A.; Maynard, J. R. J.; Butler, P. W. V.; Tizzard, G. J.; Goldup, S. M., Facial Selectivity in Mechanical Bond Formation: Axially Chiral Enantiomers and Geometric Isomers from a Simple Prochiral Macrocycle. *J. Am. Chem. Soc.* **2024**, *146* (13), 9134.

5. Savoini, A.; Gallagher, P. R.; Saady, A.; Goldup, S. M., The Final Stereogenic Unit of [2]Rotaxanes: Type 2 Geometric Isomers. *J. Am. Chem. Soc.* **2024**, *146* (12), 8472.
